# Supplementary material for: Multiple signal classification algorithm for super-resolution fluorescence microscopy
Source: Nat Commun. 2016 Dec 9;7:13752. doi: 10.1038/ncomms13752 (PMC5155148; doi:10.1038/ncomms13752)
Supplement: Supplementary Information — Supplementary Figures, Supplementary Tables, Supplementary Notes, Supplementary Methods and Supplementary References. [file ncomms13752-s1.pdf]

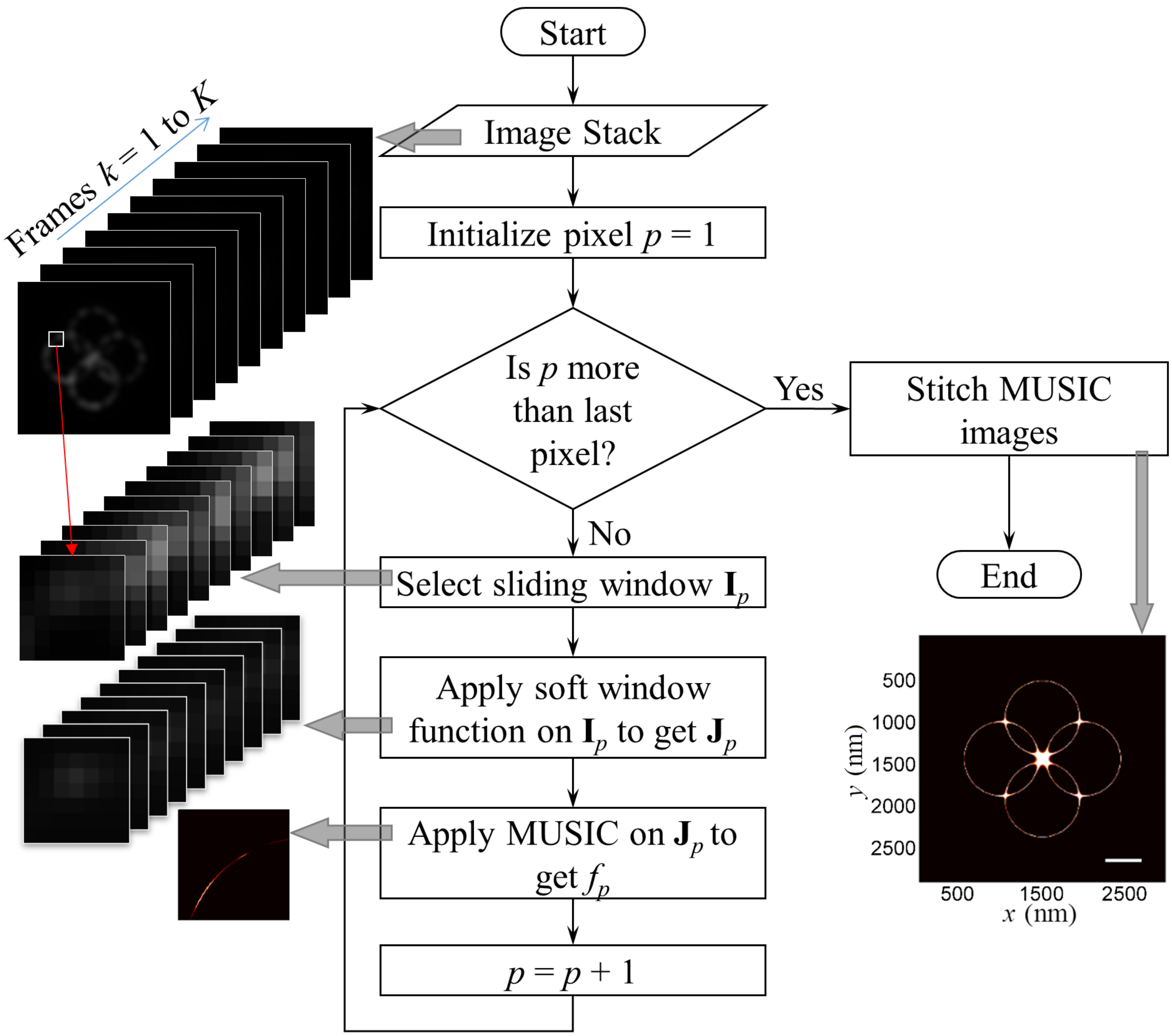

Supplementary Figure 1: **Flowchart of MUSICAL.** Additional algorithmic details appear in Supplementary Note 2.

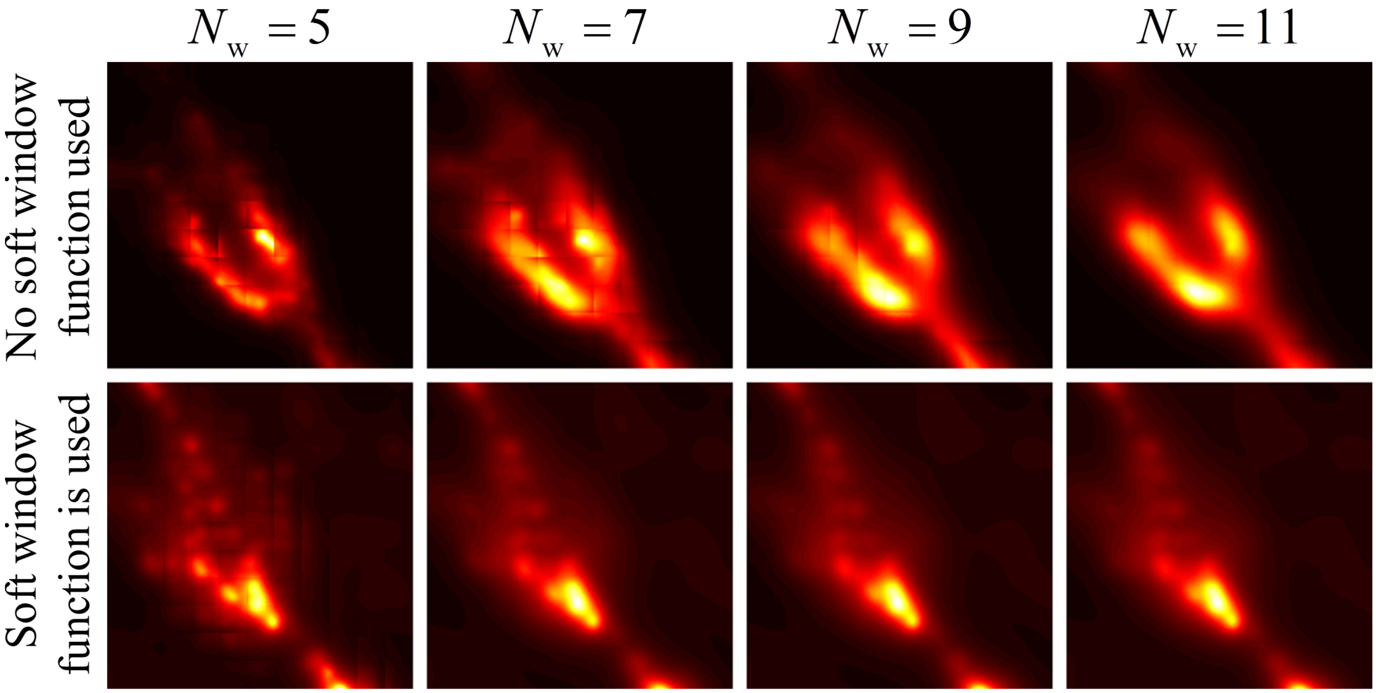

Supplementary Figure 2: **Window size and soft window.** The effect of the window size and the soft window function is demonstrated here using a small portion of region B of in-vitro sample 1. According to eq. (14), the value of N_w for in-vitro sample 1 is 7. However, the result for $N_w = 7$ without the soft window function clearly illustrates grid-like artifacts related to truncation. Moreover, the images obtained without the soft window function (top-row) show incorrect reconstruction since they do not weigh the center pixel, at which the PSF is most reliable, more than the other pixels in a window. On the other hand, use of the soft window function results in more accurate imaging result (bottom row). Further, with the use of the soft window function, values of N_w larger than recommended in eq. (14) do not change the MUSICAL result despite using more computational resources. But, the value of N_w less than recommended results in grid-like artifacts (bottom left).

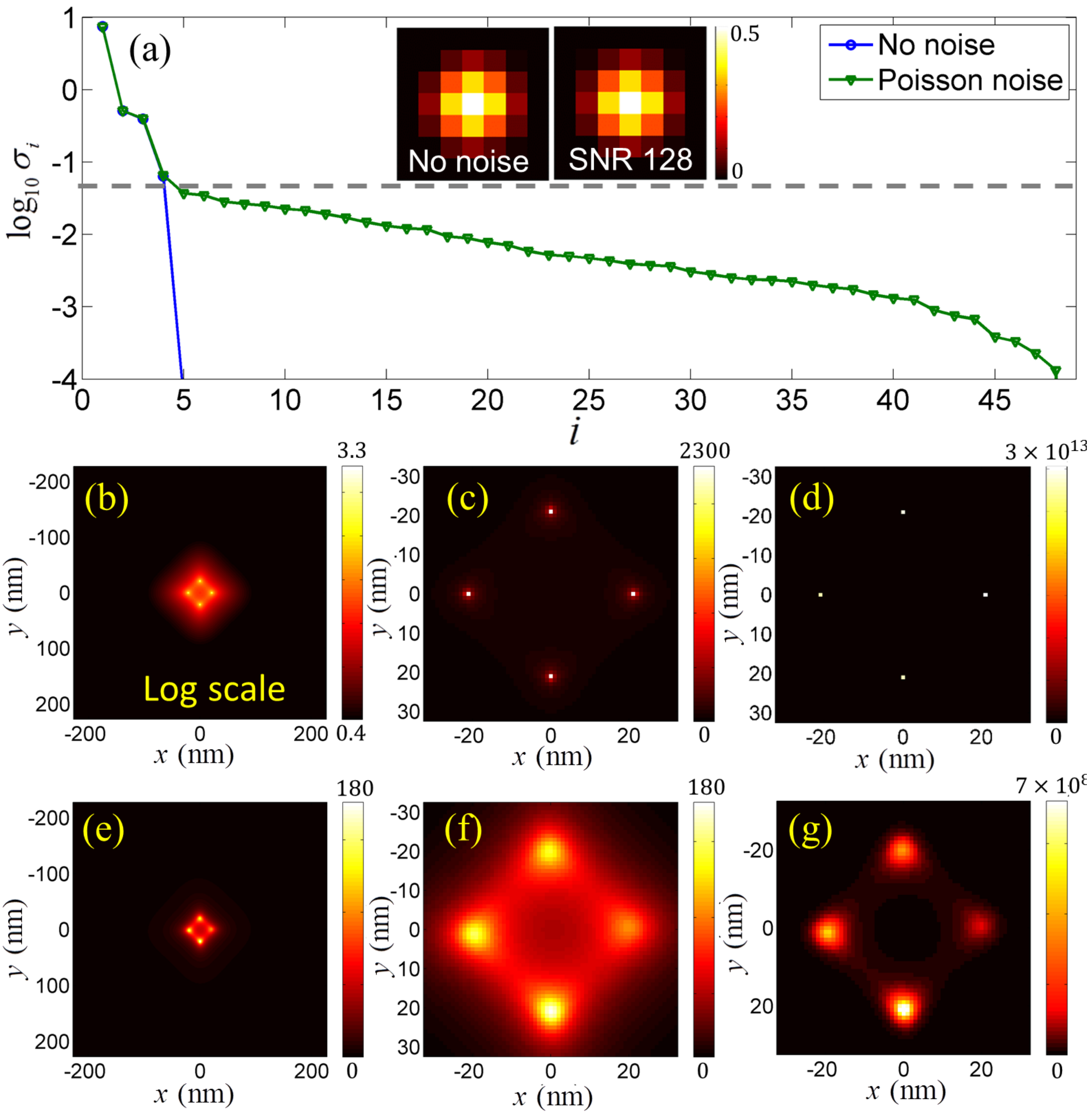

Supplementary Figure 3: **Choice of σ_0 in the condition $M < \min(N, K)$, example SynEx1.** Synthetic example SyEx1 illustrates the choice of σ_0 for the condition $M < \min(N, K)$, the effect of noise on the modified MUSIC indicator function, and the effect of the value of α. (a) Plot of singular values. Insets show the mean image of the image stacks with and without noise. Middle row corresponds to data without noise while bottom row corresponds to data with shot noise such that signal to noise ratio is 128. (b,c,e,f) correspond to $\alpha = 1$, where (c,f) are zoom-in of the central region of (b,e). (d,g) correspond to $\alpha = 4$ and show the same region as shown in (c,f). (b) is shown in logarithmic scale for ease of visualization. The color bar of (g) has been made slightly more red for the ease of visualization of the right most emitter. More details appear in Supplementary Note 4. Color bar in (b) indicates logarithm of MUSICAL indicator function values. Color bars in (c-g) indicate MUSICAL indicator function values.

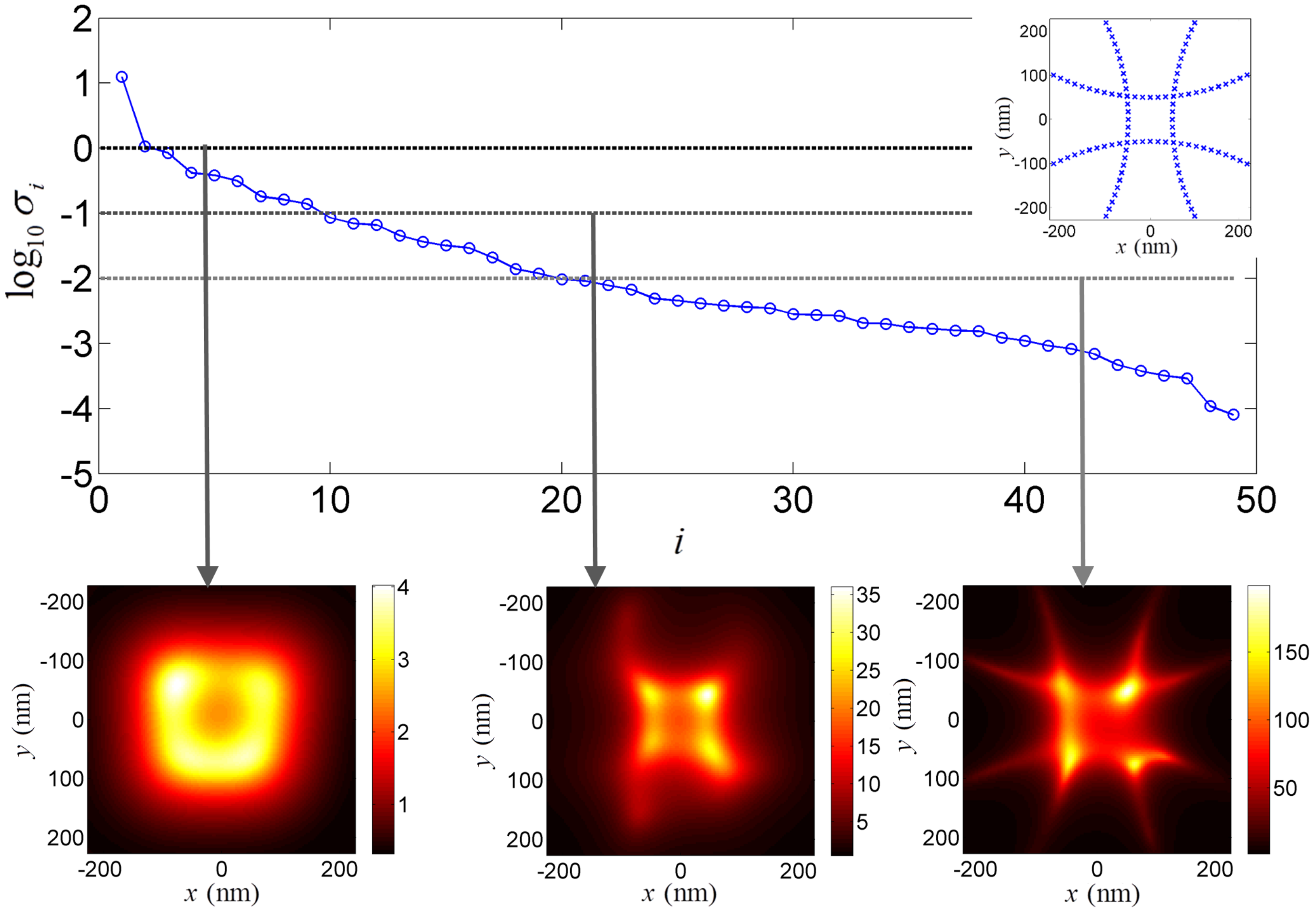

Supplementary Figure 4: **Choice of σ_0 in the condition $M \geq \min(N, K)$, example SynEx2.** Synthetic example SynEx2 in the absence of noise is used to illustrate the effect of the value of σ_0 in the condition $M \geq \min(N, K)$. Specifically, the sliding window at the center pixel of the image stack is considered. Plot of singular values is shown at the top. Inset shows the emitter distribution. Different horizontal lines correspond to different values of σ_0 and arrows from them point to the corresponding modified indicator function plots ($\alpha = 1$ is used). More details appear in Supplementary Note 4. Color bars indicate MUSICAL indicator function values.

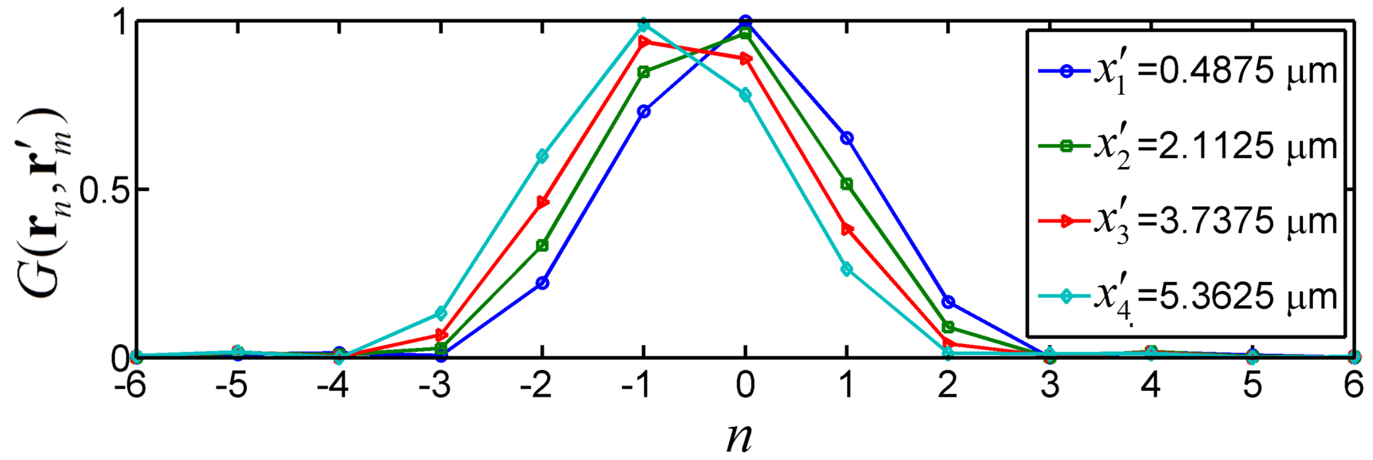

Supplementary Figure 5: **Illustration of the effect of imaging approximation on the mapping vector $G(\mathrm{r}_n, \mathrm{r}'_m)$ from a point r'_m in the sample plane to pixels r_n in the image plane.** Refer to Supplementary Note 1 and Supplementary Note 2 for its usage.

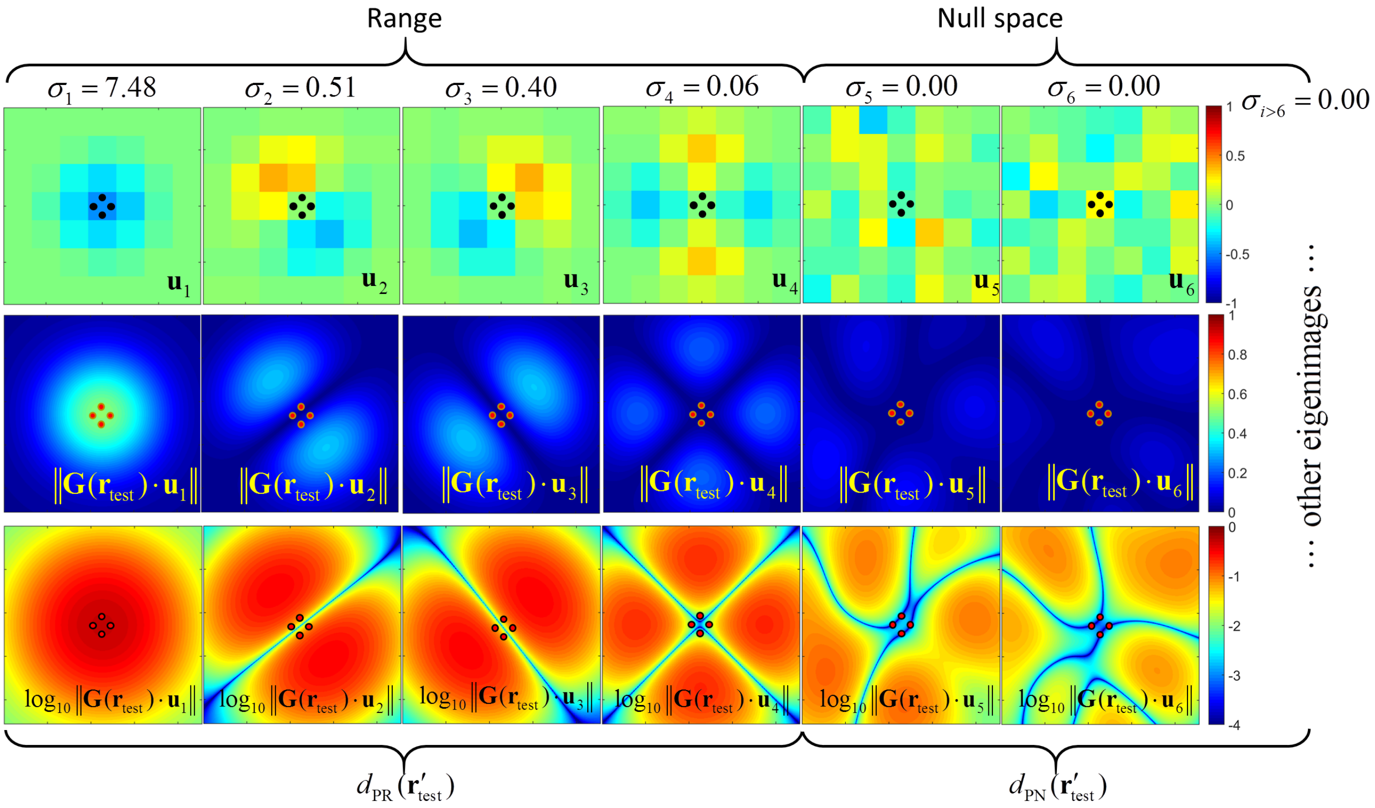

Supplementary Figure 6: **Illustration of eigenimages and projections of PSF on eigenimages.** The first six eigenimages (top row), the projections of the PSFs of the test points on the eigenimages for SynEx1 (middle row), and the logarithmic maps of the projections (bottom row) are shown here. Actual emitter locations are shown using black dots in the top row and red dots in the middle and the bottom rows. A detailed discussion is given in Supplementary Note 5. Color bars in the top row indicate the pixel values of eigenimages $\mathbf{u}_i$. Color bars in the middle row indicate the values of $\|\mathbf{G}'(\mathbf{r}'_{\text{test}})\cdot\mathbf{u}_i\|$. Color bars in the bottom row indicate the values of $\log_{10}\|\mathbf{G}'(\mathbf{r}'_{\text{test}})\cdot\mathbf{u}_i\|$.

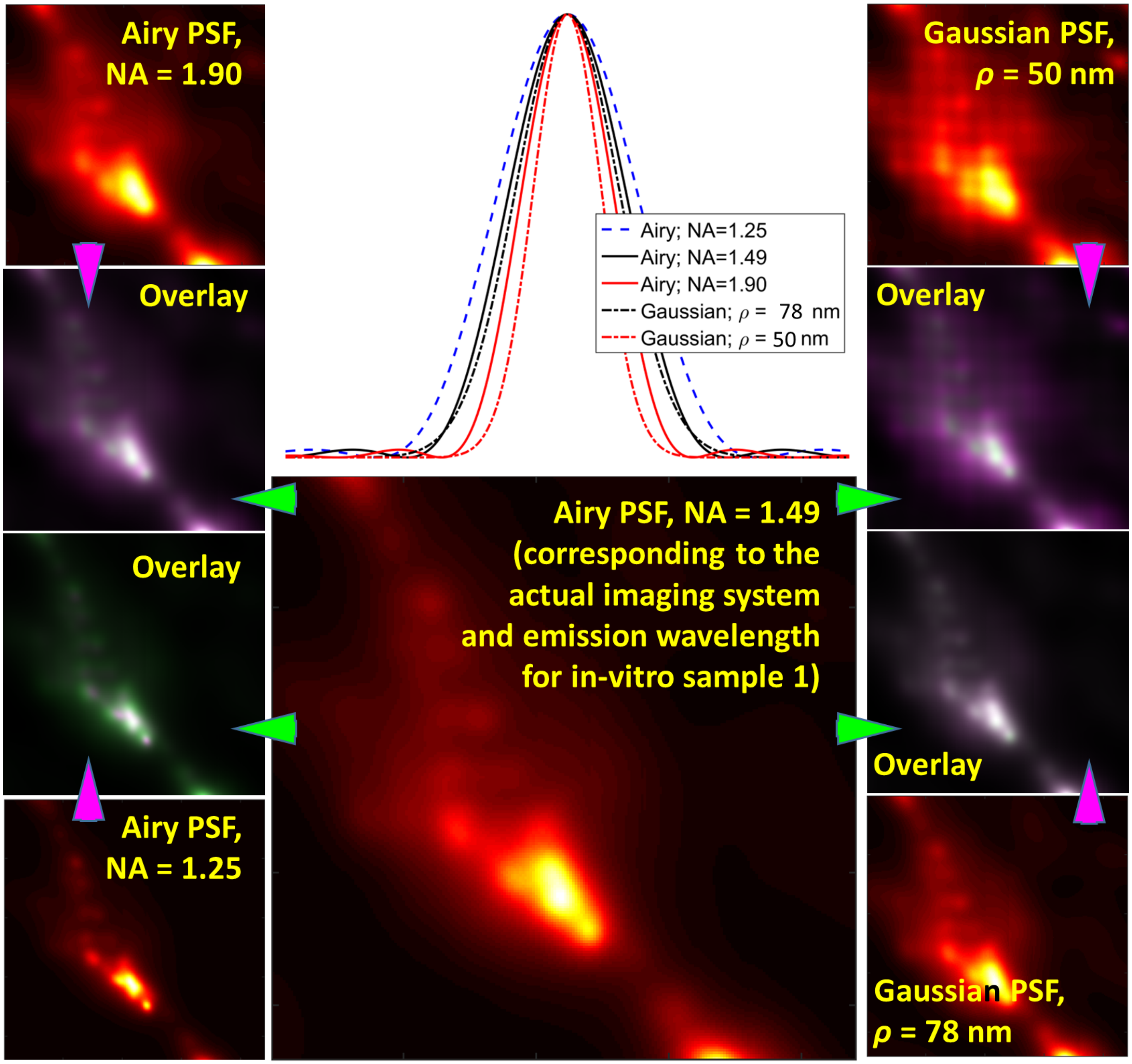

Supplementary Figure 7: **Sensitivity of MUSICAL to accurate estimation of PSF.** In case that the PSF is characterized or calibrated experimentally or does not match the actual PSF of the system, it is of interest to investigate the sensitivity of MUSICAL to the incorrect PSF. An example of sensitivity of MUSICAL to PSF is shown here using a small portion of region B of in-vitro sample 1. Images on the left side correspond to Airy PSFs with widths not matching with the actual Airy PSF while the right side corresponds to images with Gaussian approximations of PSFs. When a wider PSF is used (estimated NA smaller than actual NA), MUSICAL image appears sharper and more punctuated as seen in bottom left images. Vice versa for a narrower PSF (estimated NA larger than actual NA) as seen in top left images. In the case that the shape of the PSF is approximated as a Gaussian function, the MUSICAL result is almost the same for the actual and Gaussian approximated PSFs. However, if the Gaussian approximated PSF is too narrow, then grid-like artifacts appear in the image. In conclusion, MUSICAL is not very sensitive to the shape and width of the PSF. Nevertheless, it is significantly more sensitive to the PSF than most single molecule localization techniques and 3B. In our observation, deconSTORM also demonstrates similar sensitivity to PSF because of the sensitivity of deconvolution to the estimate of PSF.

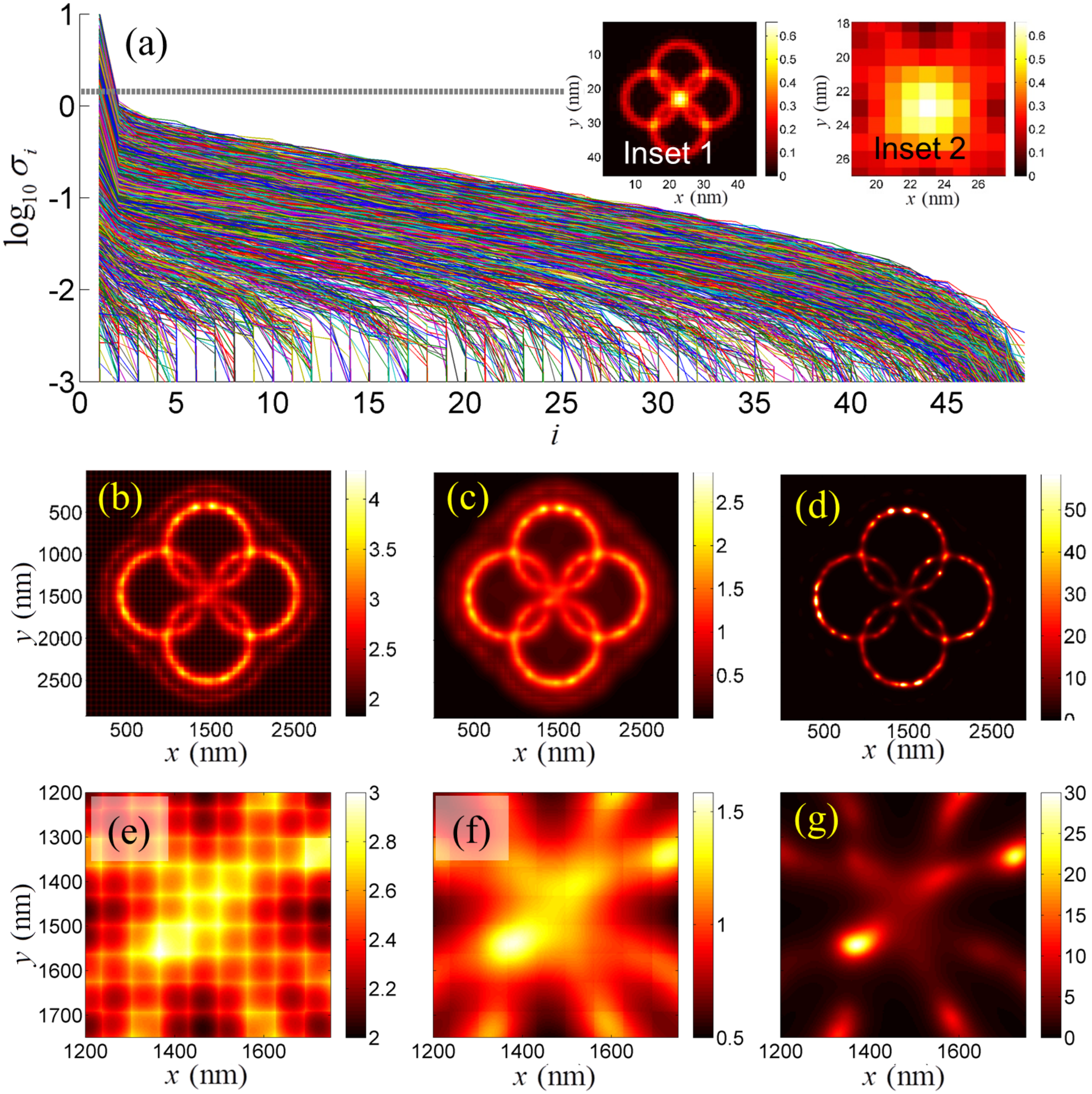

Supplementary Figure 8: **Automatic stitching ability of the modified indicator function of MUSICAL.** Synthetic example SynEx2 with noise is used to demonstrate the automatic stitching ability of the modified MUSICAL indicator function, as opposed to MUSIC's original indicator function. We consider the entire image stack with Poisson noise added to the data such that the signal to noise ratio (SNR) of the noisy image stack is 8. The singular values and the value of σ_0 (gray dashed line) are plotted in (a). σ_0 is computed using eq. (28). Further, inset 1 and inset 2 show the mean image of the entire image stack (the MUSICAL results for which are shown in (b-d)) and its central portion (the MUSICAL results for which are shown in (e-g)), respectively. (b-d) show MUSICAL results obtained using MUSIC's original indicator function (b), modified indicator function of MUSICAL with $\alpha = 1$ (c), and modified indicator function of MUSICAL with $\alpha = 4$ (d). (e-g) are the zoom-ins of the central portions of (b-d) respectively. The artifacts due to the pixel boundaries are clearly visible in (e) where the original MUSIC indicator function is used. These artifacts are significantly suppressed with the modified MUSICAL indicator function with $\alpha = 1$ as seen in (f). Further, the artifacts due to pixel boundaries are completely removed using $\alpha = 4$ (g). We have observed that in general, $\alpha \geq 2$ does not have the pixel boundary artifacts if the value of σ_0 is close to the optimal value. The halo effect seen prominently in (b,c) is due to the side lobes and is discussed in Supplementary Figure 14. The role of α is further discussed in Supplementary Note 6. Color bars in (b-g) indicate MUSICAL indicator function values.

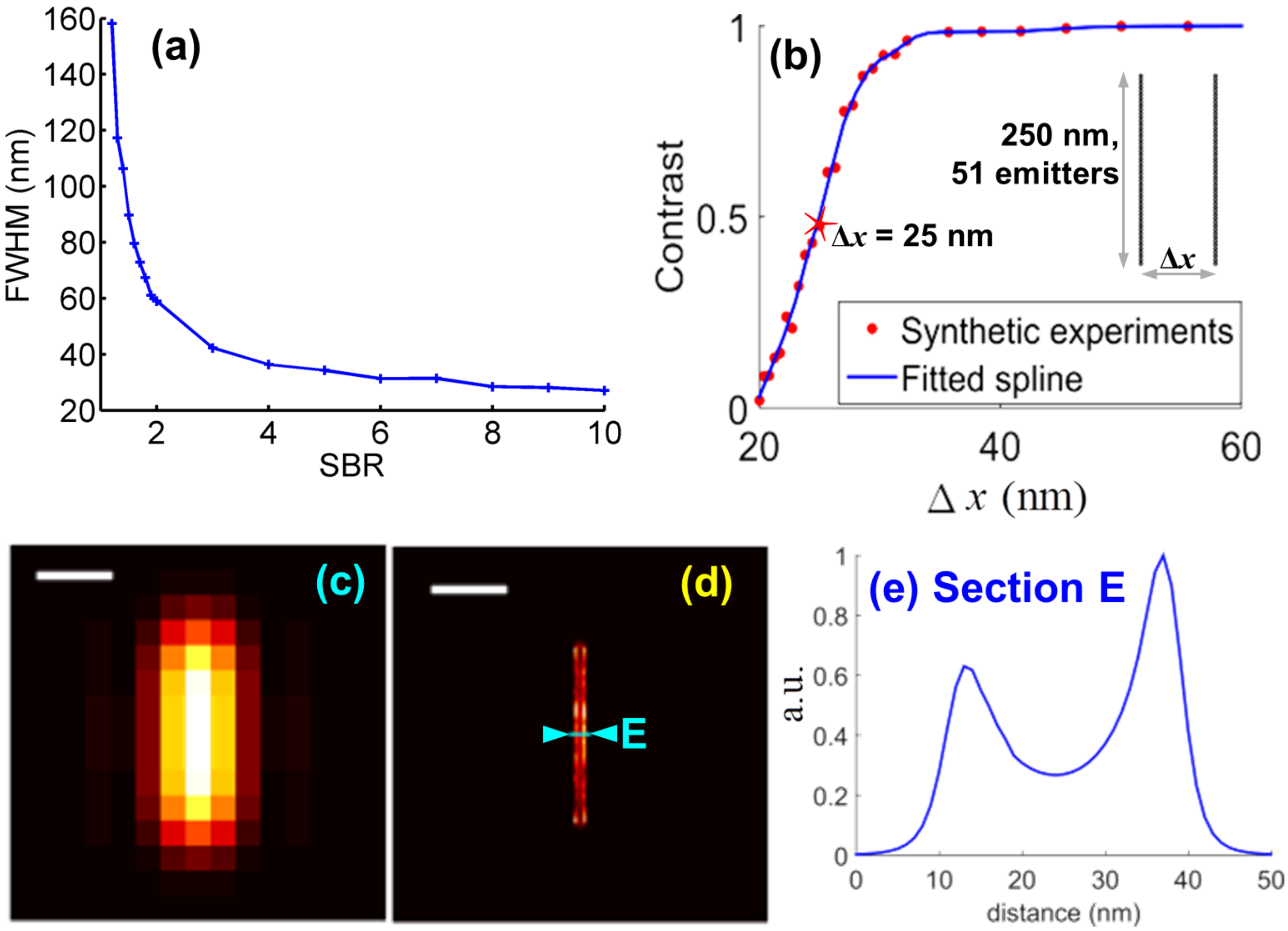

Supplementary Figure 9: **FWHM and structural resolution of MUSICAL.** FWHM for SynEx3 as a function of the signal to background ratio (SBR) is shown in (a). The minimum value of FWHM is 27.11 nm for SBR 10 and the maximum value is 158.2 nm for SBR 1.2. Quantification of structural resolution through synthetic example SynPairDelX is done in (b-e). The contrast used here is defined as $\frac{I_{\max}-I_{\min}}{I_{\max}+I_{\min}}$ where $I_{\max}$ is the maximum intensity among the two consecutive maxima and $I_{\min}$ is the intensity of the minimum between them. (b) shows the contrast between two lines of emitter separated by distance Δx computed using synthetic dataset SynPairDelX. The geometry of SynPairDelX is shown in the inset. The red colored star shown on the plot in (b) corresponds to $\Delta x = 25$ nm and has a contrast of 0.48. The mean image for the image stack and MUSICAL result for the SynPairDelX data with $\Delta x = 25$ nm are shown in (c,d), respectively. The intensity at section E shown in (d) is plotted in (e). It shows that the maxima are clearly separated with a distance of 25 nm between them. No other statistical method could resolve these two lines. STORM localized only three emitters for this data. Scale bars: (c,d) 200 nm.

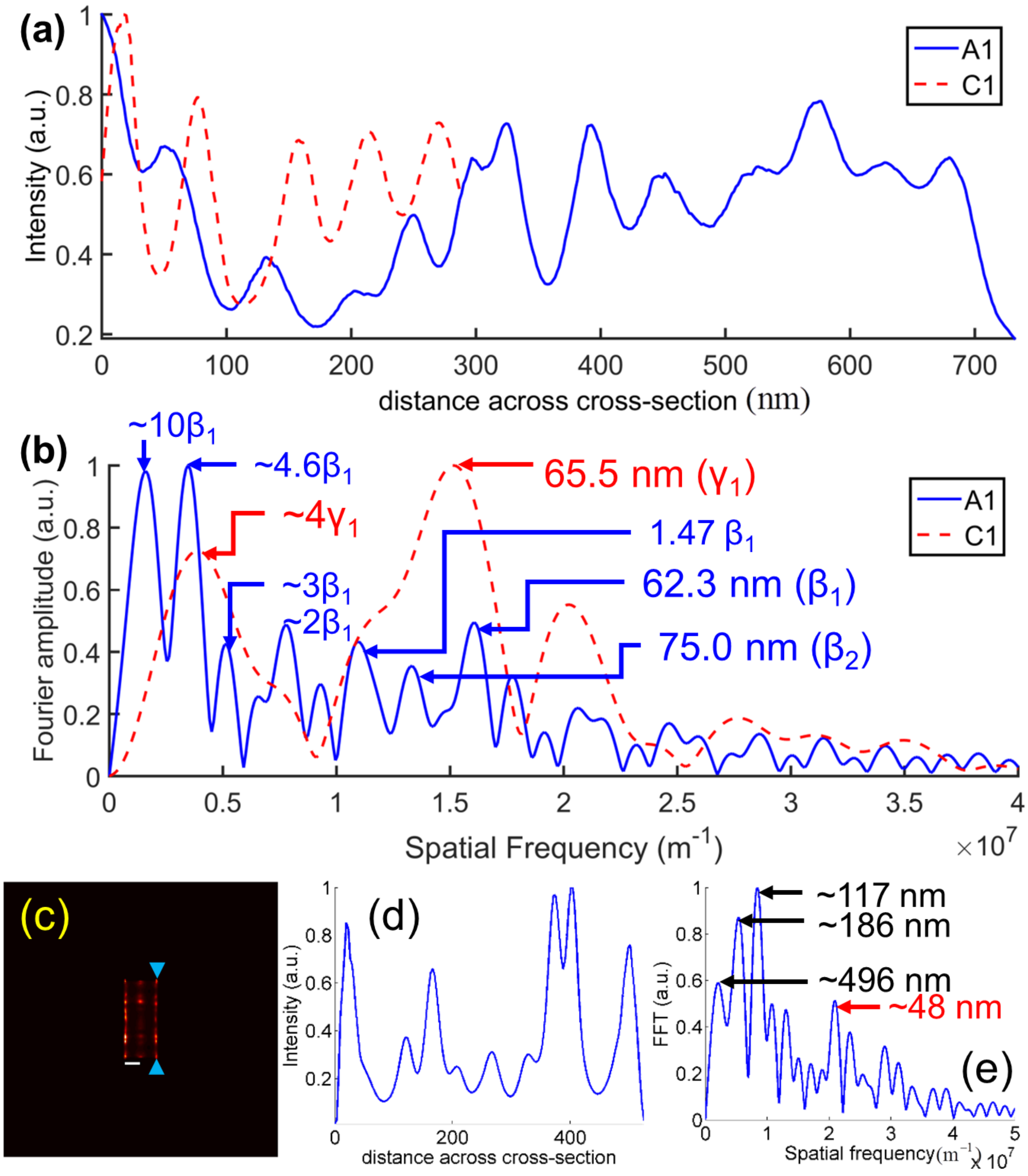

Supplementary Figure 10: **Periodicity observed in actin filaments.** MUSICAL intensity profiles of lines A1 and C1 in Fig. 1 of the main paper are shown in (a). Fourier spectra of these are shown in (b). For computing the Fourier amplitudes, the intensity profiles were mean compensated and fast Fourier transform was computed. As noted in (b), there are two peaks in the Fourier spectrum for the profile A1 at sampling frequencies corresponding to 62.5 nm (β_1) and 75 nm (β_2). The other peaks in the Fourier spectrum of A1 are harmonics of these two sampling frequencies. Similarly, there is one clear peak in the Fourier spectrum for the profile C1 at sampling frequency corresponding to 65.5 nm (γ_1) and the other peak is the 4th order harmonic of this frequency. We use SynPeriod to further validate the periodicity observed. MUSICAL result for SynPeriod is shown in (c). (d) shows the MUSICAL intensity along the section indicated by the blue arrows in (c). (e) shows its Fourier spectrum. The Fourier spectrum in (e) shows dominant peaks at about 50 nm or its multiples. This indicates that the peaks observed in Fourier spectra of the in-vitro actin filaments are related to the periodicity of actin filaments. Scale bar: (c) 100 nm.

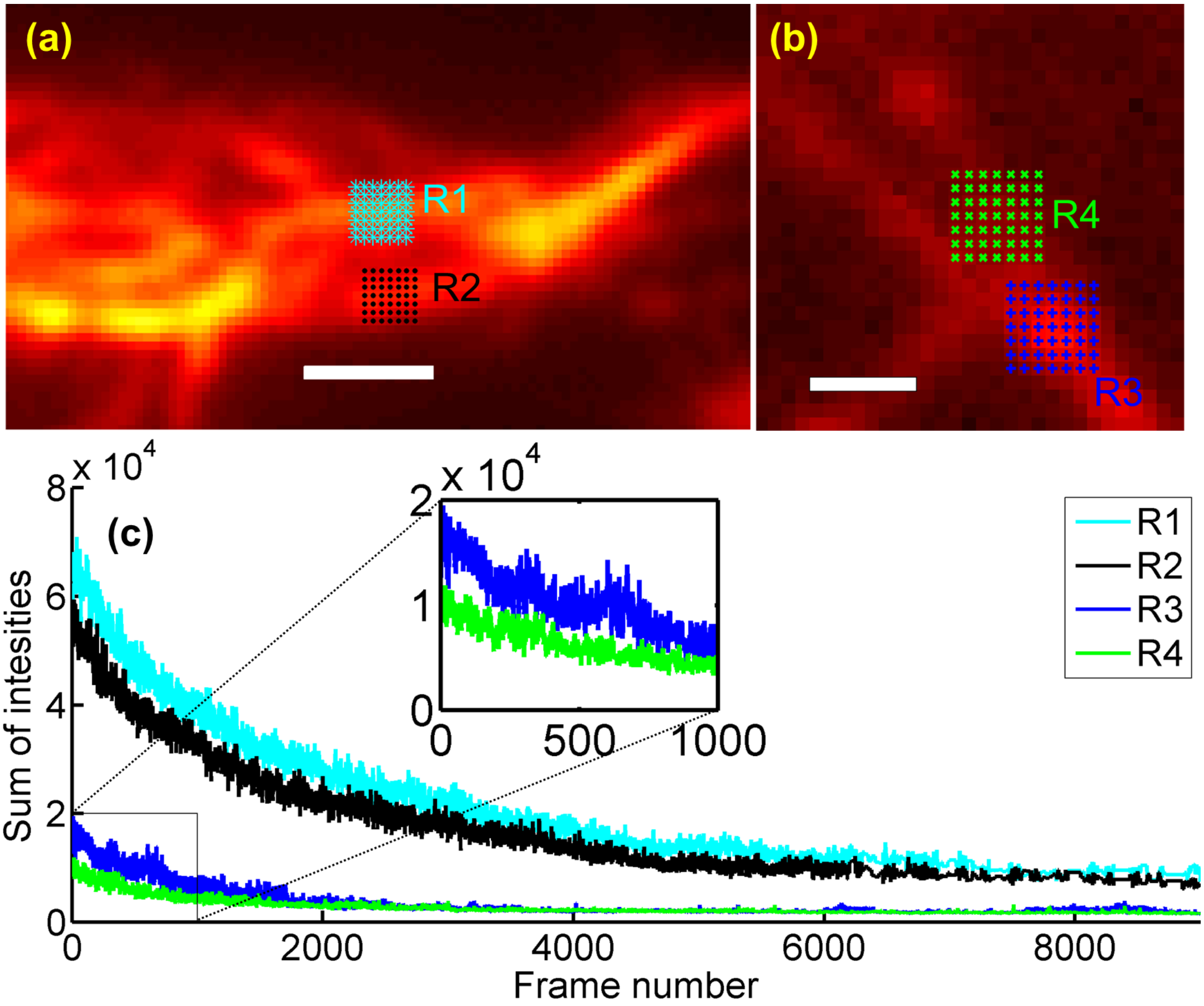

Supplementary Figure 11: **Comparison of density of fluorophores in in-vitro samples 1 and 2.** Plots of sum of the measured intensities in different 7×7 regions R1 and R2 of sample 2 (a) and R3 and R4 of sample 1 (b) are shown in (c). The plotted intensities are an indicator of density of fluorophores in the region since all the remaining experimental factors are the same. The intensities in sample 2 (R1 and R2) are significantly larger than in sample 1 (R3 and R4), indicating much higher density of fluorophores in sample 2 than in sample 1. For the same distribution of on and off times, higher density of fluorophores translates to less sparse blinking. The regions R3 and R4 correspond to the branch of actin filaments in region A of sample 1 and a single actin filament, respectively. It is seen in the inset of Supplementary Figure 11(c) that the intensity in R3 is approximately 1.8 times the intensity in R4, indicating more densely packed fluorophores in R3. All the plots correspond to image stacks captured with excitation laser's intensity of 205.6 W cm^{-2}. Scale bars: (a) 1 μm; (b) 500 nm.

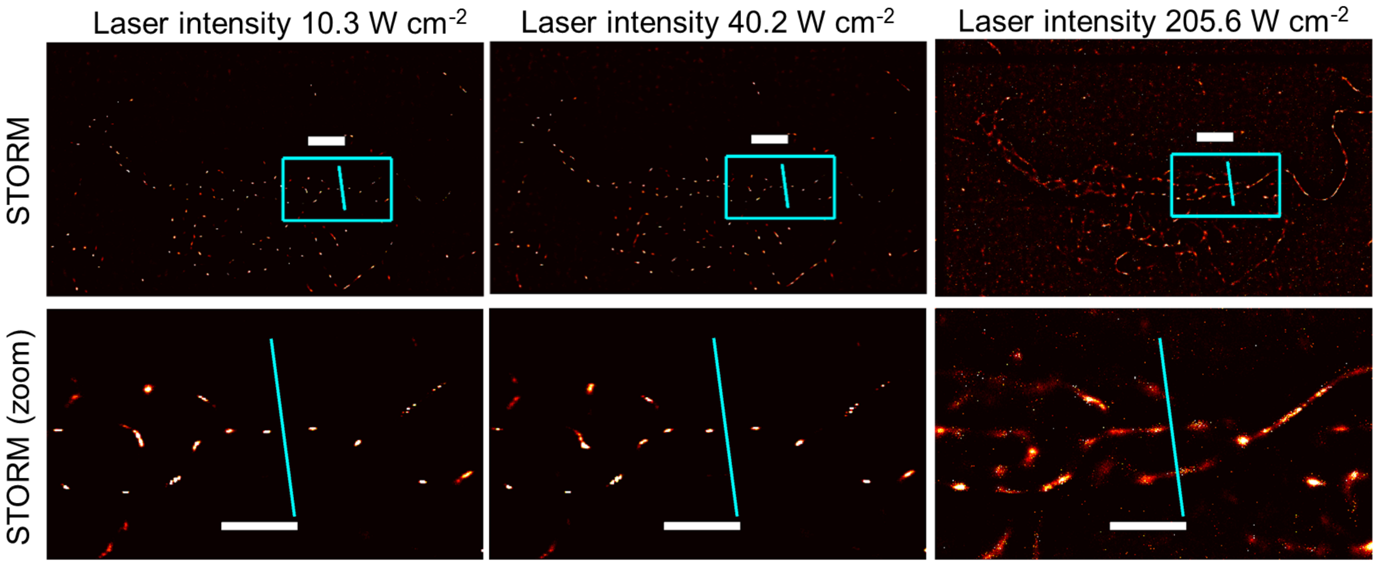

Supplementary Figure 12: **STORM results for in-vitro sample 2.** The outline of the zoom-in regions and the section line have the same coordinates as Fig. 2 of the main paper. The details in STORM results are significantly less than the details in the MUSICAL results in Fig. 2 of the main paper. As inferred in Supplementary Figure 11, the blinking in sample 2 is not sparse even for 205.6 W cm^{-2} and thus is not well-suited for STORM. Unsurprisingly, MUSICAL outperforms STORM for this example. Scale bars: (a-c) 2 μm; (d-f) 1 μm.

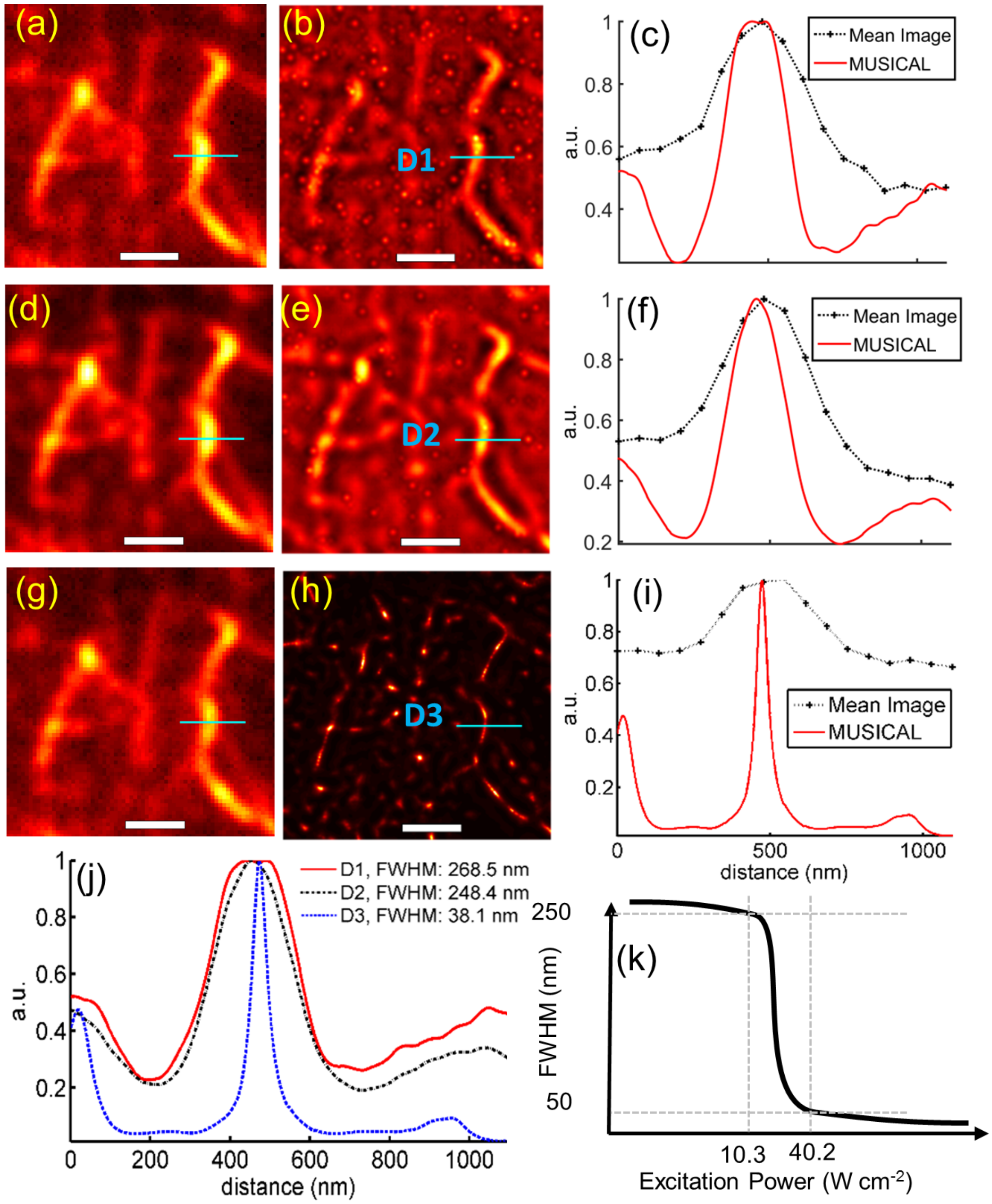

Supplementary Figure 13: **Results for in-vitro sample 3 imaged at different excitation powers.** Actin filaments are imaged using a TIRF microscope and an excitation laser with power 0.93 W cm^{-2} (a-c, top row), 10.3 W cm^{-2} (d-f, second row), and 205.6 W cm^{-2} (g-i, third row). The mean images are shown in (a,d,g), while the MUSICAL results are shown in (b,e,h). Intensities in the mean image and the MUSICAL image for cross-sections D1-D3 are compared in (c,f,i), respectively. Comparison of MUSICAL for sections D1-D3 is shown in (j). Empirical plot of power versus MUSICAL FWHM is shown in (k). More details appear in Supplementary Note 7. Scale bars: 1 μm.

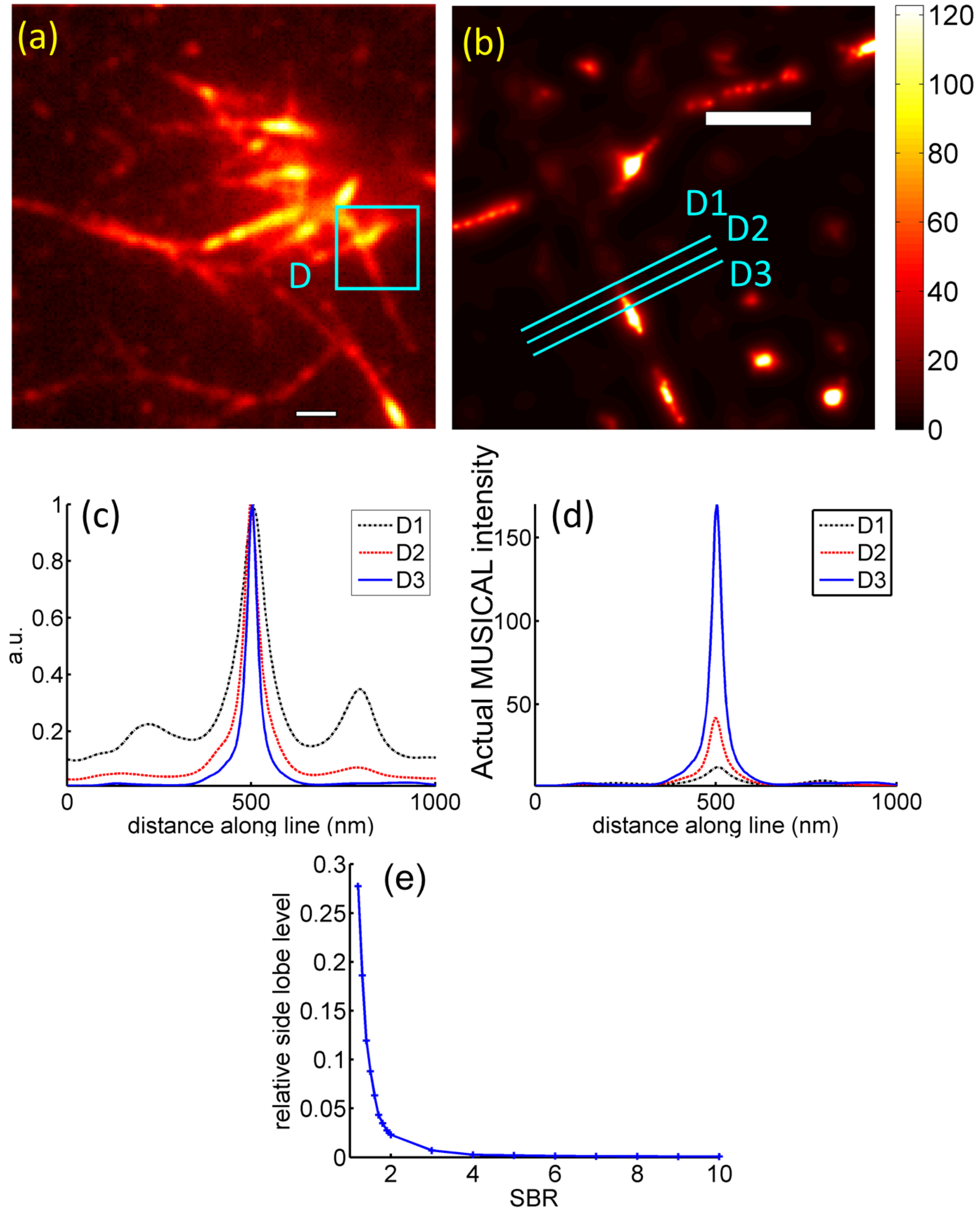

Supplementary Figure 14: **MUSICAL and side lobes.** Mean image of in-vitro sample 1 and the region D are shown in (a). MUSICAL result for region D is shown in (b). Normalized MUSICAL intensity profiles from sections D1-D3 are shown in (c) and the corresponding actual intensities are shown in (d). The presence of slide lobes is clearly evident in (c). We note that this effect is similar to the well-known effect of increased side lobes when pupil filters such as annular filters are introduced to reduce the size of the focal spot of a focusing system [1]. Analogously, the improvement of resolution in MUSICAL as compared to the original image stack is accompanied with side lobes. However, as noted in (d), the level of side lobes remains almost the same for all the three curves, irrespective of their intensities. Further, we plot the side lobe levels relative to the peak intensity of MUSICAL result ($\alpha = 4$) as a function of the SBR for SynEx3 in (e). For low values of SBR, the side lobe levels may be quite prominent. We also note that such side lobes appear in other results in electromagnetic imaging problem as well [2], although their occurrence was not discussed. In practice, this effect of side lobes can be removed by adjusting the minimum intensity of the color bar to a small non-zero value commensurate with the side lobe levels. More sophisticated approaches, such as discussed in Supplementary Note 9, may be adopted. Scale bars: (a) 1 μm, (b) 500 nm. Color bar in (b) indicates MUSICAL indicator function values.

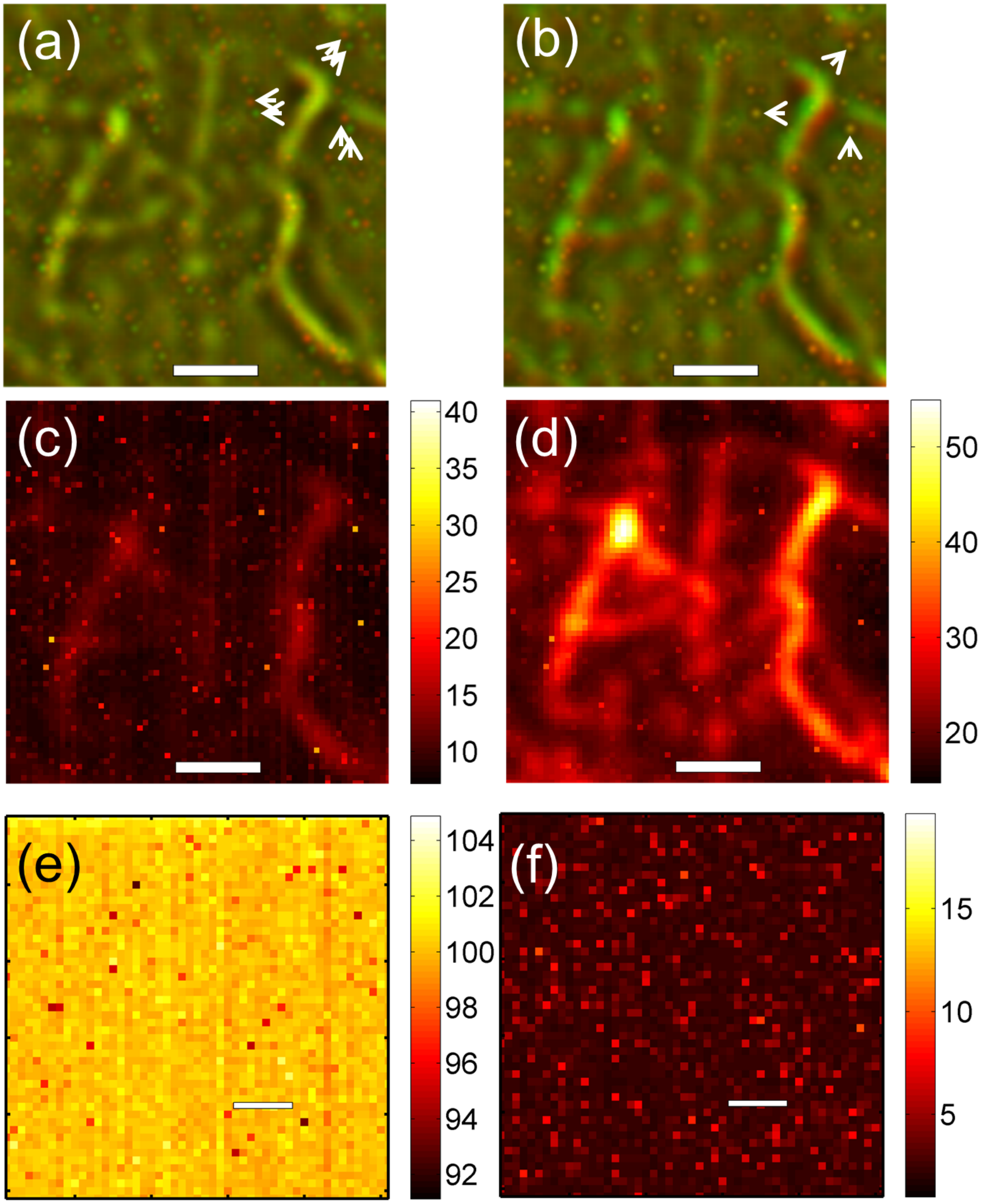

Supplementary Figure 15: **MUSICAL and sensitivity to the camera noise.** MUSICAL results for sample 3 acquired using laser intensities 0.93 W cm^{-2} and 10.3 W cm^{-2} are fused as red and green channels respectively and shown here. (a) shows the result with the samples aligned while (b) shows the fusion of MUSICAL image without sample alignment.(c,d) Standard deviations's map for the measured intensities using laser intensities 0.93 W cm^{-2} and 10.3 W cm^{-2}, respectively. (e,f) Mean and standard deviations, respectively, of the measured intensities in a random image region of scMOS sensor measured without any laser excitation. More details appear in Supplementary Note 8. Scale bars: (a-d) 1 μm; (e,f) 500 nm. Color bars in (c,d,f) indicate standard deviation of pixel intensity values. Color bar in (e) indicate mean value of pixel intensity values.

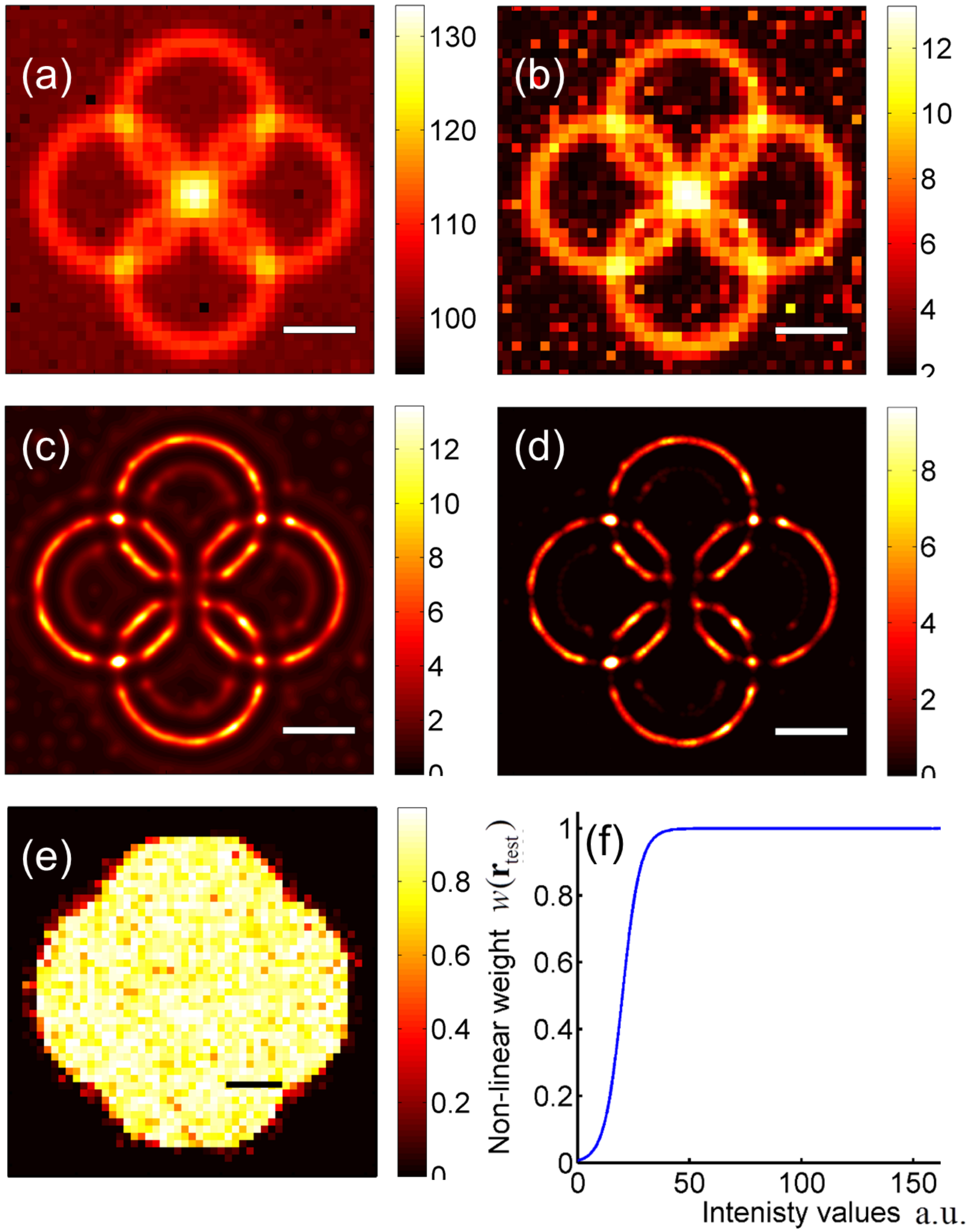

Supplementary Figure 16: **Compensation for camera noise and suppression of side lobes.** Results for synthetic example SynEx4Bck are shown here. (a,b) Mean and standard deviations, respectively, of the image stack. (c) MUSICAL result. (d) MUSICAL results with compensation of noise and suppression of side lobes using the schemes in Supplementary Note 9. (e) $\text{scale}(p)$ computed using eq. (30). (f) $w(\mathbf{r}_{\text{test}})$ computed using eq. (31). Scale bars: 500 nm. Color bars: (a) mean of pixel intensity values; (b) standard deviation of pixel intensity values; (c,d) MUSICAL indicator function values; (e) values of $\text{scale}(p)$.

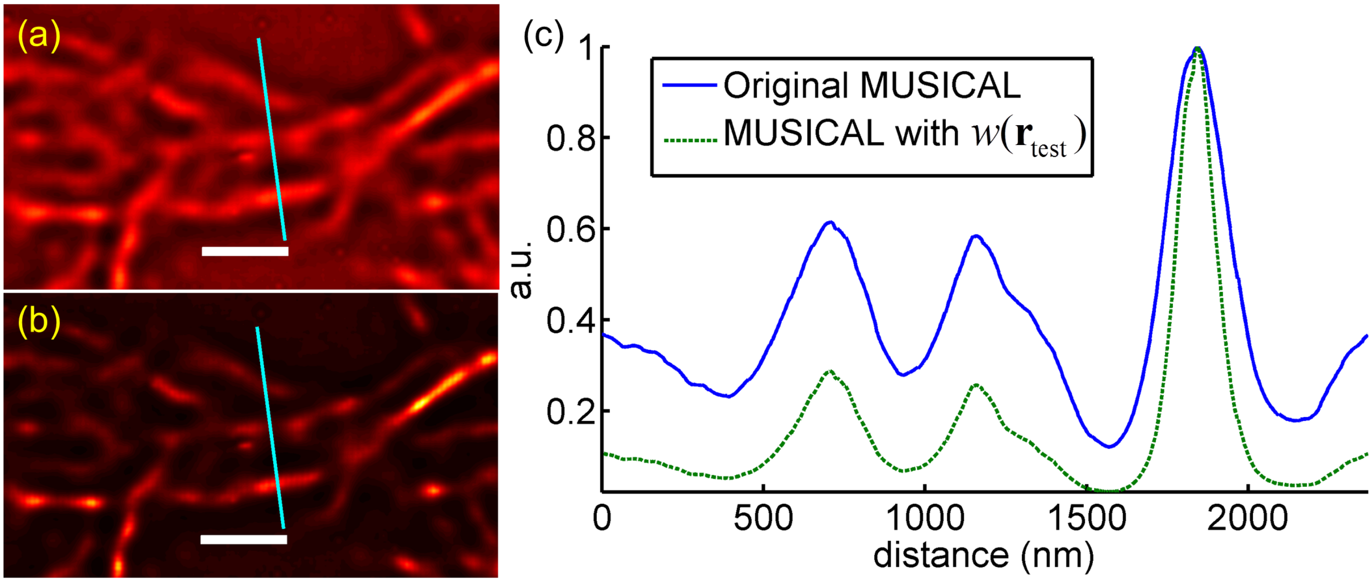

Supplementary Figure 17: **Result of side lobes suppression for in-vitro sample 2.** Reduction of background using $w(\mathbf{r}_{\text{test}})$ of Supplementary Note 9 in the MUSICAL result for sample 2. (a) zoom-in section of MUSICAL result for sample 2, the same as Fig. 2(m) of the main paper. (b) the same section, but with the incorporation of $w(\mathbf{r}_{\text{test}})$. (c) comparison of cross-sections. Scale bars: 1 μm.

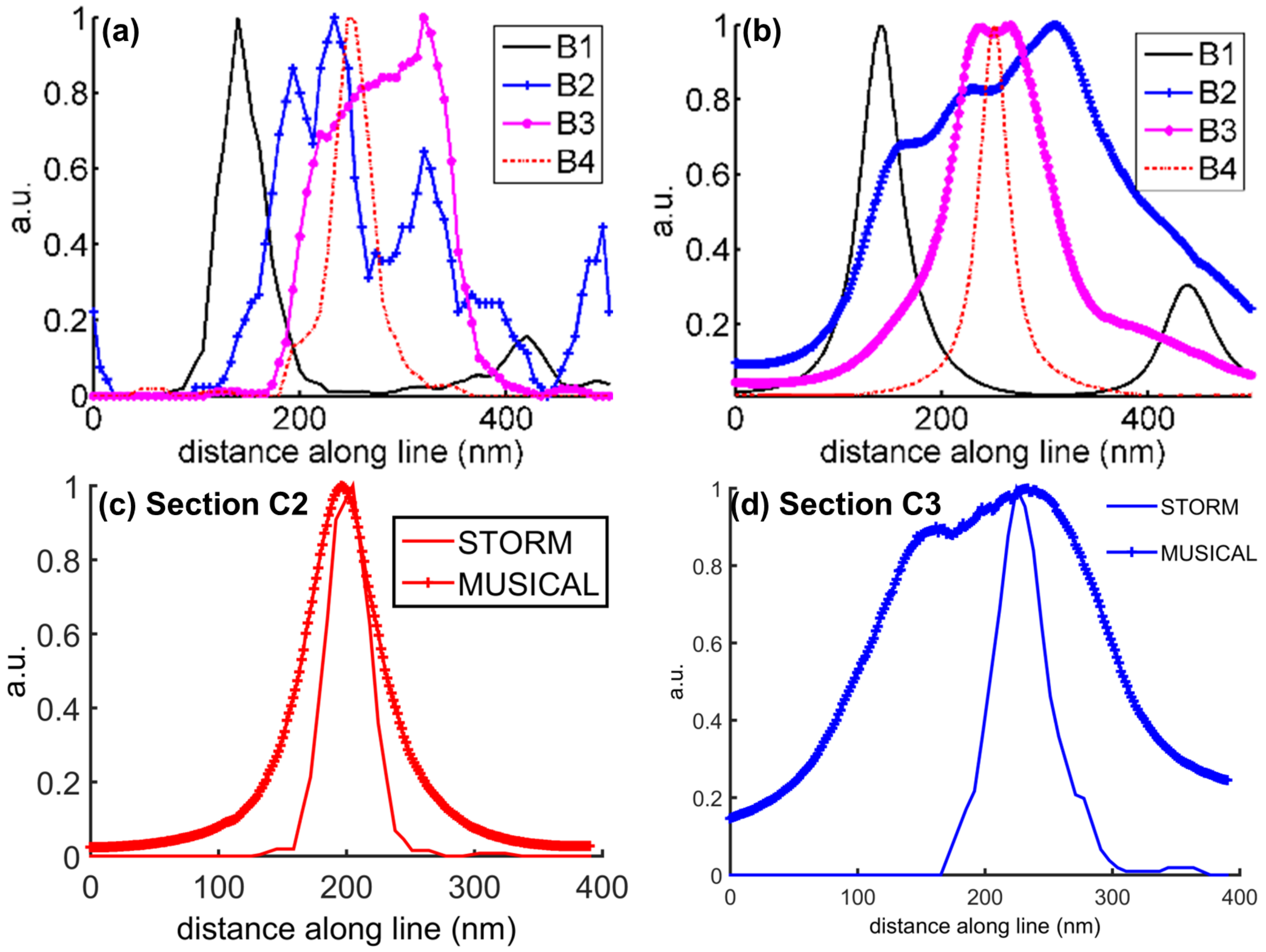

Supplementary Figure 18: **Comparison between MUSICAL and STORM for in-vitro sample 1.** STORM intensity profiles at cross-sections B1-B4 in Fig. 1(f) are shown in (a). MUSICAL intensity profiles at cross-sections B1-B4 in Fig. 1(g) are shown in (b). STORM and MUSICAL intensity profiles at cross-section C2 in Fig. 1(i,j) are shown in (c). STORM and MUSICAL intensity profiles at cross-section C3 in Fig. 1(i,j) are shown in (d). Discussion on this figure appears in Supplementary Note 10.

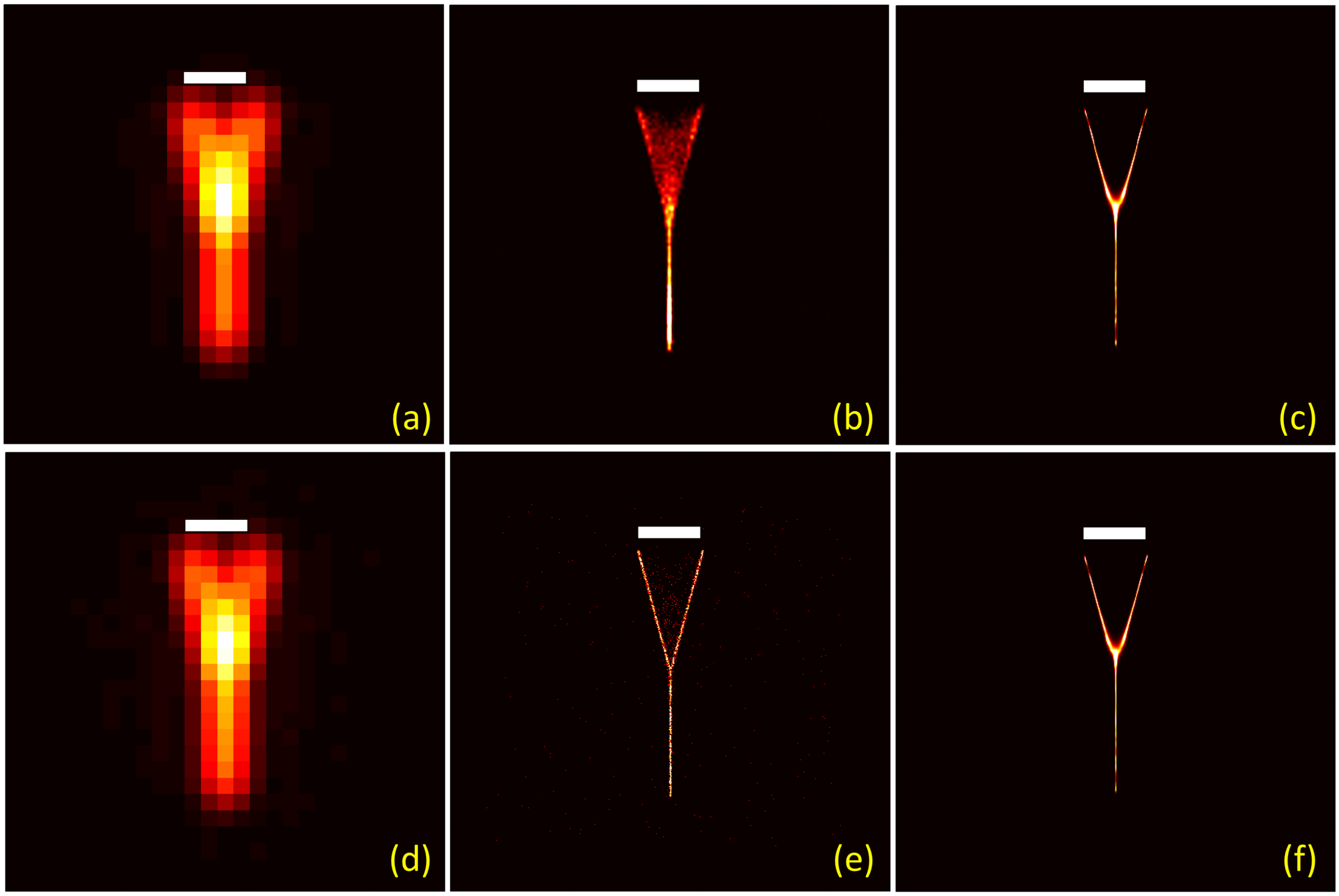

Supplementary Figure 19: **Comparison between MUSICAL and STORM using synthetic forks similar to Fig. 1(g).** Results for synthetical experiments SynFork1 and SynFork2 which simulate a fork similar to Fig. 1(g). Top row —SynFork1;Bottom row –SynFork2. (a,d) mean image of 1,000 frames. (b,e) STORM result (c,f) MUSICAL result. Discussion on this figure appears in Supplementary Note 10. Scale bars: 250 nm.

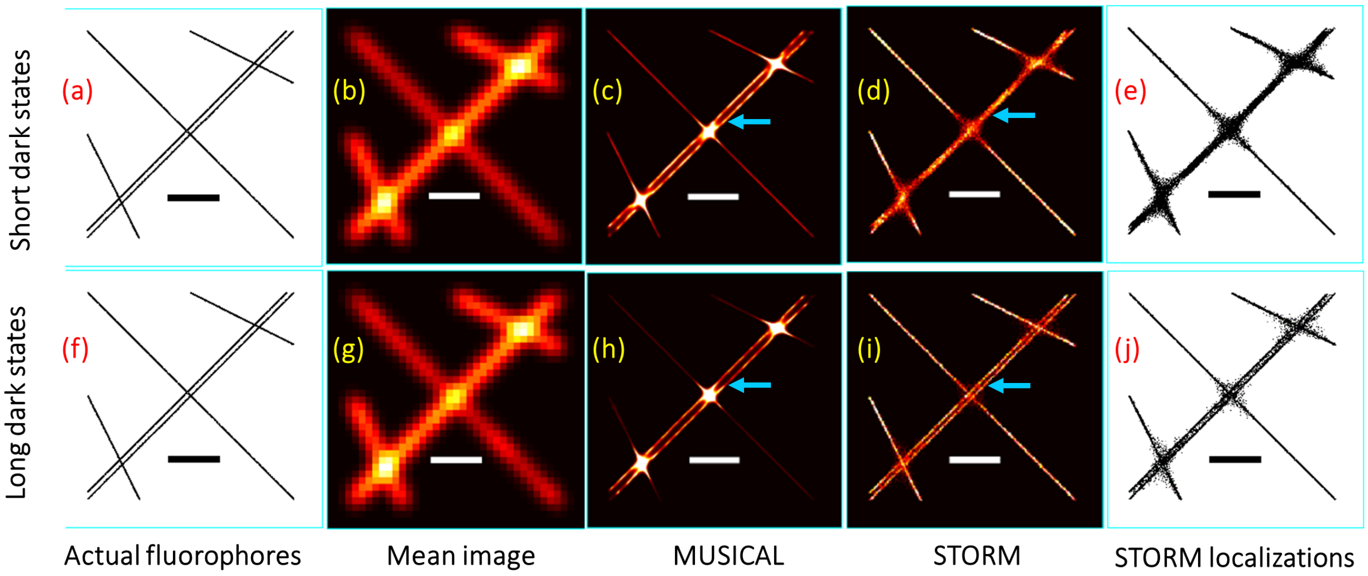

Supplementary Figure 20: **Comparison between MUSICAL and STORM using synthetic example SynSTORM.** Synthetic example SynSTORM is used to compare MUSICAL and STORM. (a-e) correspond to fluorophores with short dark states while (f-j) correspond to fluorophores with long dark states. (a,f) show actual emitter profiles. (b,g) show mean images of the image stacks. (c,h) show MUSICAL result. (d,i) show STORM images and (e,j) show the points localized by STORM. Discussion on this figure appears in Supplementary Note 10. Scale bars: 500 nm.

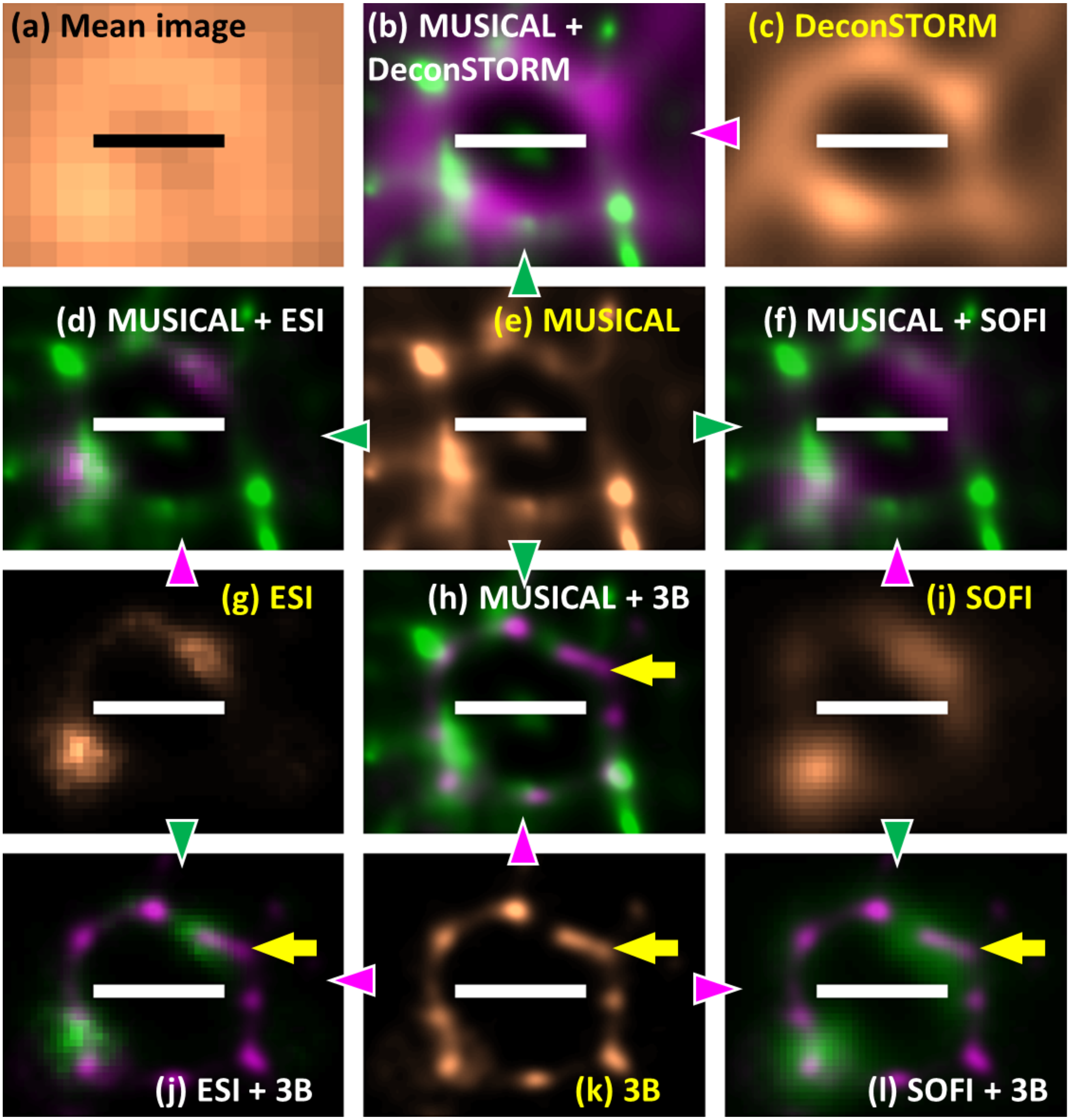

Supplementary Figure 21: **Comparison between MUSICAL and other statistical analysis method for the 3B test data provided at [3].** Imaging results of different methods and overlay of different pairs of methods are given above. It is seen that the shape details are better constructed by 3B (k) than any other method including MUSICAL. However, we noted that the vertex of the polygon reconstructed by 3B highlighted using yellow arrows in (h,j-l) does not agree with the results of the other methods. We are not sure if it is due to a local minimum in the iterative optimization of 3B or it is an accurate location of the vertex. Scale bars: 500 nm.

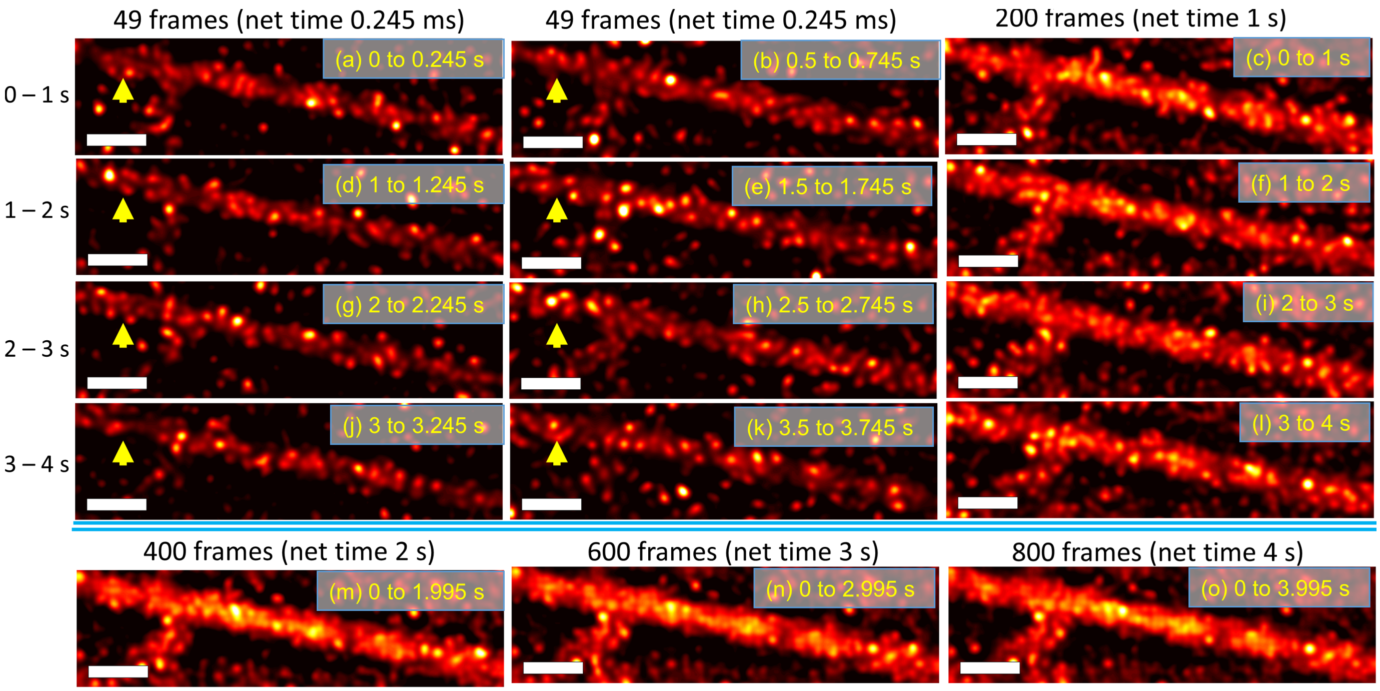

Supplementary Figure 22: **Time lapse study for live-cell microtubules sample 1 and influence of the number of frames used in MUSICAL.** We use region B of live-cell microtubules sample 1 shown in Fig. 5 to discuss the benefit of reducing the number of frames for live-cell studies involving dynamic molecules. MUSICAL images obtained with different numbers of frames and in different time slots of the imaging time-line are shown above. The same lower bound criterion (0.4 times of the 99.9% of the histogram of MUSICAL intensities) is used for all the images. MUSICAL results in the first two columns of the top panel are significantly sharper than the remaining MUSICAL results. This is because the blurring effect due to the local dynamics in the vicinity of the microtubule can be alleviated by using less frames. Further, the background is relatively less cluttered in MUSICAL images with 49 frames. Higher numbers of frames imply collection of more signal from spatially more spread traces of freely diffusing labeled molecules, and thus a more pronounced background. The bottom pane showing MUSICAL results for increasing number of frames indicates that the structural details reconstructed by MUSICAL start saturating at sufficiently large number of frames. Lastly, we note that the images in the first two columns of the top panel, each using 49 frames, do not have overlapping time windows. Unsurprisingly, they differ from each other in the structural details. This indicates the utility of MUSICAL in time lapse studies, allowing direct visualization of dynamics on small time scales. In summary, using the smallest possible number of time frames allows for sharp, less cluttered, super-resolved images with the potential of time lapse studies. Scale bars: 500 nm.

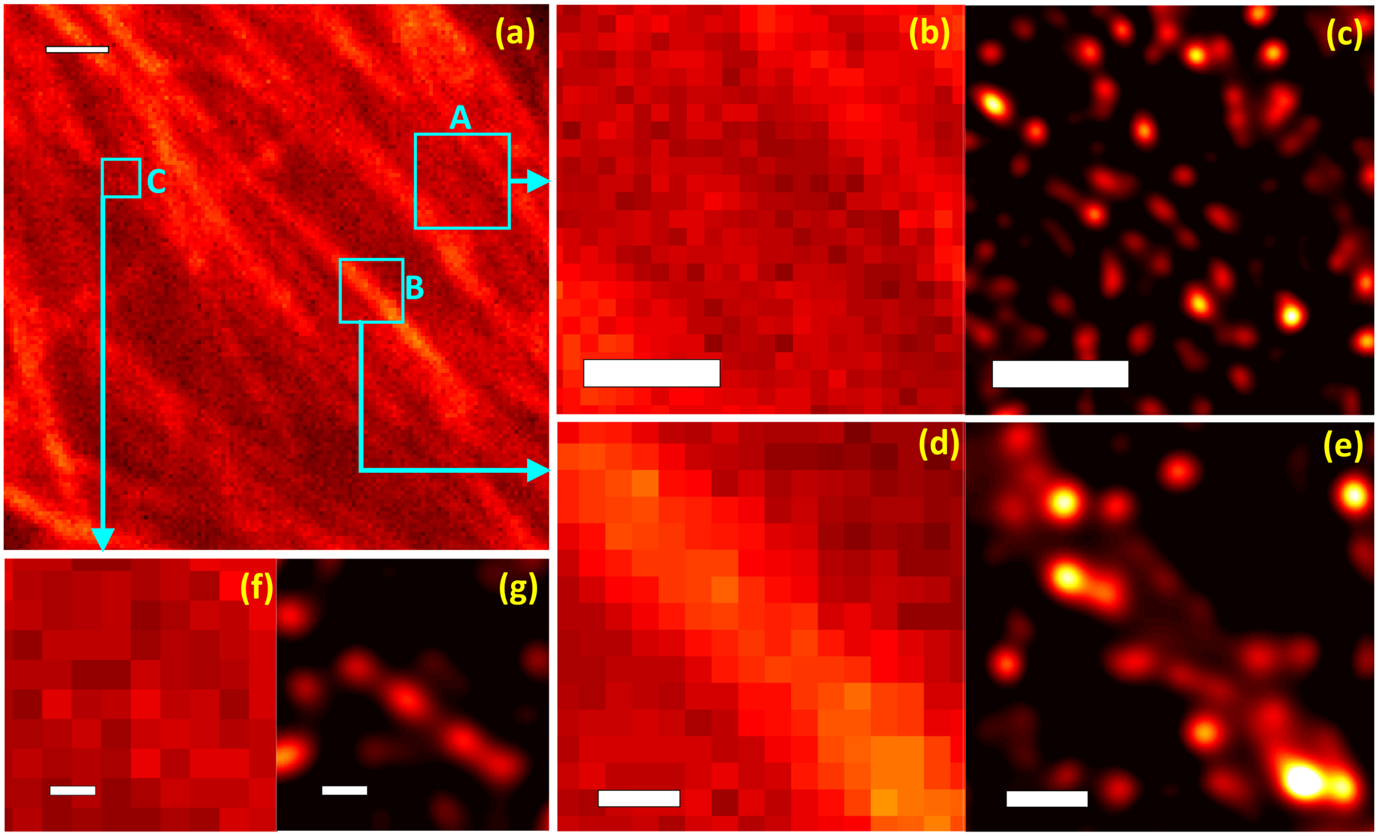

Supplementary Figure 23: **MUSICAL results for live-cell microtubules sample 2, generated using 49 frames spanning 49 ms of time.** Live-cell microtubule sample 2 is imaged at a frame rate of 1,000 frames per second, i.e. 1 ms per frame. (a) shows the mean image of 49 frames of the complete sample. (b,c) show mean and MUSICAL images of the region A. (d,e) show mean and MUSICAL images of the region B. and (f,g) show mean and MUSICAL images of the region C. It is seen that even in this sample, MUSICAL reconstructs sub-100 nm details. The lower bound for this example is chosen heuristically to be 0.30 times the 99.9% of the histogram of MUSICAL intensities. The lower bound was decreased in the light of the smaller spatial spread of diffusing molecules captured in 49 ms, as opposed to sample 1, where 49 frames correspond to 245 ms. Scale bars: (a) 1 μm; (b,c) 500 nm; (d,e) 200 nm; (f,g) 100 nm.

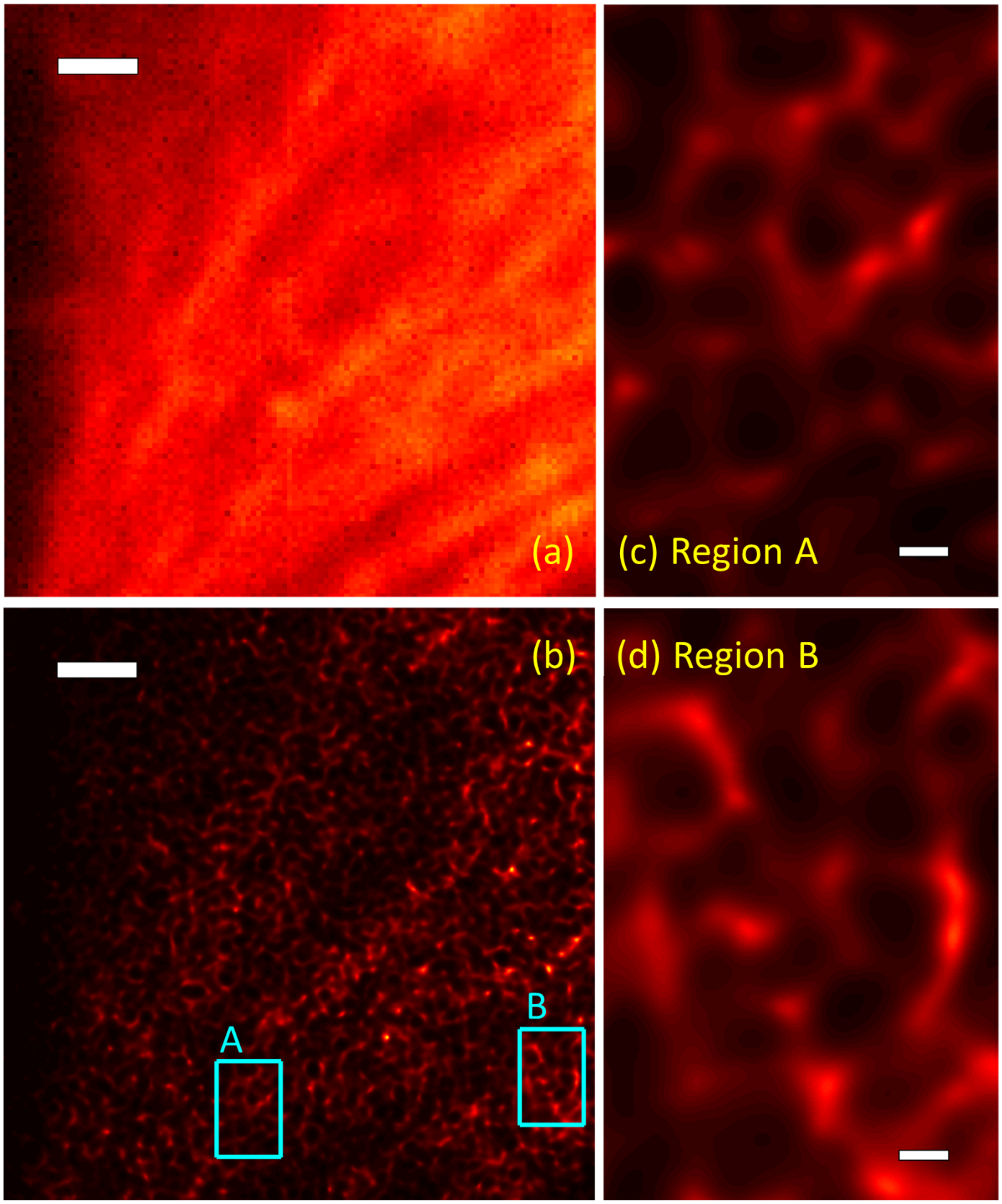

Supplementary Figure 24: **Result of MUSICAL for the live-cell cortical-cytoskeletal F-actin.** The live-cell imaging of actin cytoskeleton was performed in a cell line stably expressing Lifeact-GFP. The whole cytoskeleton is subject to fluorescence labeling. However, due to the TIRF illumination geometry, mainly the cortical actin cytoskeleton was imaged. The cells were illuminated by a 488 nm laser, excitation power of 25 W cm^{-2}, and imaged with 1 ms exposure. The MUSICAL result shown was generated using 100 frames only, which corresponds to 100 ms. Mean image of 100 frames (a) has significantly less details than the corresponding MUSICAL image (b). Zoom-ins of regions A and B are shown in (c,d), respectively. The results are in good agreement with the known structures of the cortical F-actin mesh [4], [5]. Notably the geometry as well as the mesh size agrees well with Fig. 3(g,h) of [4] and Fig. 6(d) of [5]. Scale bars: (a,b) 1 μm and (c,d) 100 nm.

Technique	Instrumentation dependence	Dye dependence	Number of frames	Frame rates (fps)	Excitation Power	Spatial view region	Resolution
STED	Yes	Yes	1	$\approx$ 10-100	High	Unlimited	$\approx$ 20 nm
STORM	No	Yes	$\geq$1000	$\approx$ 10-100	High	Unlimited	20-30 nm
3B	No	No	100-1000	#	Medium	100 to 10,000 pixels	$\approx$ 50 nm
SOFI	No	No	$\geq$100	#	Medium	Unlimited	$\approx$ 100 nm
ESI	No	No	$\geq$100	#	Medium	Unlimited	$\approx$ 80 nm
MUSICAL	No	No	$\geq$50	#	Medium	Unlimited	30-50 nm

Supplementary Table 1. Qualitative comparison of super-resolution techniques. # Limited by blinking statistics. More discussion appears in Supplementary Note 11.

Sample	Dye	No. of washes*	No. of frames	Frame rate (fps)	Laser Intensity (W cm^{-2})
In-vitro Sample 1	Phalloidin Atto 565	3	10,000	200	205.6
In-vitro Sample 2	Phalloidin Atto 565, tetraspeck beads (40 nm diameter)	5	10,000	200	10.3, 40.2, 205.6
In-vitro Sample 3	Phalloidin Atto 565	0	20,000	100	0.93, 10.3, 205.6
Live-cell microtubules Sample 1	Lifeact GFP	not applicable	1,000	200	25
Live-cell microtubules Sample 2	Lifeact GFP	not applicable	49	1,000	25
Live-cell F-actin	Lifeact GFP	not applicable	100	1,000	25

Supplementary Table 2. Details of samples are provided here. * Number of washes indicate the number of times the sample is washed before introducing the imaging buffer.

SUPPLEMENTARY NOTE 1. CONCEPT OF MUSICAL

We first begin with the mathematical model of the temporal image stack formed by blinking fluorophores. Then, the core concept of MUSICAL is presented.

Mathematical model of the image of blinking fluorophores

For the ease of reference, we denote points in the image plane as $\mathbf{r}$ and the sample plane as $\mathbf{r}'$. For simplicity, we assume that contribution from off-plane emitters can be ignored, such that the sample can be considered two-dimensional. The x and y coordinates (i.e. lateral coordinates) of the points in the image plane are represented as (x, y). Similarly, the lateral coordinates of the points in the sample plane are represented as (x', y'). Necessary subscripts may be added as relevant.

Let there be M emitters (individual blinking fluorophores) located at $\mathbf{r}'_m, m = 1$ to M in the sample plane of a microscopy system. We denote the temporal blinking state of the mth emitter as a binary signal $b_m(t)$, its emission strength as e_m, and the emission wavelength as λ.

Let the image acquisition rate be given by Δt^{-1} frames per second, where Δt is the image acquisition time per frame. The total emission from the emitter during the kth frame is given by

$$s_m(k) = e_m \int_{(k-1)\Delta t}^{k\Delta t} b_m(t)\,\mathrm{d}t. \tag{1}$$

In the imaging system, we denote the centers of the regularly arranged pixels in the image plane as $\mathbf{r}_n; n = 1$ to N where N is the number of pixels. The pixel is assumed to be square of width w. We interpret the point spread function (PSF) $G(\mathbf{r}, \mathbf{r}')$ of the imaging system as a mapping which maps the emission from a point $\mathbf{r}'$ in the sample plane to the intensity at a point $\mathbf{r}$ in the image plane. The intensity measured at the nth pixel is given by

$$I_k(\mathbf{r}_n) = \int\limits_{\left(y_n - \frac{w}{2}\right)}^{\left(y_n + \frac{w}{2}\right)} \int\limits_{\left(x_n - \frac{w}{2}\right)}^{\left(x_n + \frac{w}{2}\right)} \sum_{m=1}^{M} G(\mathbf{r}, \mathbf{r'}_m) s_m(k) \mathrm{d}x \mathrm{d}y\ , \tag{2}$$

We assume a diffraction limited imaging system in which the dimensions of pixels are significantly smaller than the extent of the main lobe of the PSF of the system. For an imaging system whose PSF can be approximated as an Airy disk, this implies that $w \ll 1.22\lambda/\mathrm{NA}$, where NA is the numerical aperture of the imaging system. Then, using the approximation

$$\int\limits_{\left(y_n - \frac{w}{2}\right)}^{\left(y_n + \frac{w}{2}\right)} \int\limits_{\left(x_n - \frac{w}{2}\right)}^{\left(x_n + \frac{w}{2}\right)} G(\mathbf{r}, \mathbf{r'}_m) \mathrm{d}x \mathrm{d}y \approx G(\mathbf{r}_n, \mathbf{r}'_m) w^2, \tag{3}$$

the intensity at the nth pixel can be simplified as

$$I_k(\mathbf{r}_n) = \sum_{m=1}^{M} w^2 G(\mathbf{r}_n, \mathbf{r}'_m) s_m(k). \tag{4}$$

We consider a simple example to illustrate the nature of $G(\mathbf{r}_n, \mathbf{r}'_m)$, where $\mathbf{r}_n$ are the centers of the discrete pixels in the image space but the emitter locations $\mathbf{r}'_m$ are not restricted to discrete grid points. The PSF of most incoherent imaging systems can be represented using an Airy disk pattern

$$G(\mathbf{r}, \mathbf{r}') = \left(\frac{\mathrm{J}_1\left(\frac{2\pi \mathrm{NA}}{\lambda \mathrm{M}} |\mathbf{r} - \mathrm{M}\mathbf{r}'|\right)}{|\mathbf{r} - \mathrm{M}\mathbf{r}'|} \right)^2 , \tag{5}$$

where M is the optical magnification of the imaging system and $\mathrm{J}_1()$ represents the Bessel function of 1st order and 1st kind. Let us assume and imaging system with magnification 1 and NA=1.49. Emission wavelength of $\lambda = 510$ nm is assumed. Let us consider one-dimensional case where $y = 0$ and $y' = 0$. In Supplementary Figure 5, we plot the values of $G(\mathbf{r}_n, \mathbf{r}'_m)$ for $x_n = 6.5n\ \mu\mathrm{m}$, where integer n varies from -7 to 7 and four values of x'_m, all of which correspond to the same pixel area (namely $n = 0$). Thus, while $G(\mathbf{r}, \mathbf{r}')$ is a point-to-point mapping, $G(\mathbf{r}_n, \mathbf{r}')$ is a point-to-pixel mapping. MUSICAL uses the point-to-pixel mapping $G(\mathbf{r}_n, \mathbf{r}')$.

Intensity measurements in the kth frame at all the pixels can be written as a matrix equation as follows:

$$\mathbf{I}_k = \mathbf{G}\mathbf{s}_k\ , \tag{6}$$

where,

$$\mathbf{I}_k = \left[\ I_k(\mathbf{r}_1) \quad I_k(\mathbf{r}_2) \quad \ldots \quad I_k(\mathbf{r}_N)\ \right]^{\mathrm{T}}\ , \tag{7}$$

$$\mathbf{G} = w^2 \left[\ \mathbf{G}'(\mathbf{r}'_1) \quad \mathbf{G}'(\mathbf{r}'_2) \quad \ldots \quad \mathbf{G}'(\mathbf{r}'_M)\ \right]\ , \tag{8}$$

$$\mathbf{G}'(\mathbf{r}') = \left[\ G(\mathbf{r}_1, \mathbf{r}') \quad G(\mathbf{r}_2, \mathbf{r}') \quad \ldots \quad G(\mathbf{r}_N, \mathbf{r}')\ \right]^{\mathrm{T}}\ , \tag{9}$$

$$\mathbf{s}_k = \left[\begin{array}{cccc} s_1(k) & s_2(k) & \ldots & s_M(k) \end{array}\right]^{\mathrm{T}} , \tag{10}$$

and the superscript ^{T} denotes vector or matrix transpose. For the ease of further reference, we refer to $\mathbf{G}'(\mathbf{r}'_m)$, i.e. mapping from a location *where an emitter is actually present*, as the emitter mapping vector (EMV). This is to differentiate the EMV $\mathbf{G}'(\mathbf{r}'_m)$ from the more general vector $\mathbf{G}'(\mathbf{r}')$.
Lastly, the intensity measurements from all the frames can be collected together as a 2-dimensional matrix, in which each row corresponds to a pixel and each column corresponds to a frame. The complete image stack which contains images from time frames $k = 1\,\mathrm{to}\,K$ can be written as a matrix

$$\mathbf{I} = [\mathbf{I}_1 \quad \mathbf{I}_2 \quad \ldots \quad \mathbf{I}_K]\,. \tag{11}$$

The core concept of MUSICAL
The core concept of MUSICAL revolves around the mathematical range of the matrix $\mathbf{I}$ and its connection to the actual physical system and sample that created the observed intensities in the matrix $\mathbf{I}$ (thus called the physical definition of the range of the matrix). We discuss this concept below.

Physical definition of the range of the matrix $\mathbf{I}$ — It is evident from eqs. (6,11) that each vector in $\mathbf{I}$ is a linear combination of the EMVs $\mathbf{G}'(\mathbf{r}'_m)$, $m = 1$ to M. Thus, the range of the matrix $\mathbf{I}$ is spanned by the EMVs $\mathbf{R} : \{\mathbf{G}'(\mathbf{r}'_m); m = 1\,\mathrm{to}\,M\}$.

Mathematical definition of the range of the matrix $\mathbf{I}$ — Singular value decomposition (SVD) of the matrix $\mathbf{I}$ gives the spatial basis vectors $\mathbf{u}_{\sigma_i}$ and temporal basis vectors $\mathbf{v}_{\sigma_i}$ with associated singular values σ_i, such that $\mathbf{I}\,\mathbf{v}_{\sigma_i} = \sigma_i \mathbf{u}_{\sigma_i}$. The range of the matrix $\mathbf{I}$ is thus given by $\mathbf{R} : \{\mathbf{u}_{\sigma_i \neq 0}\}$. The vectors $\mathbf{u}_{\sigma_i}$ are referred to as eigenimages and simply written as $\mathbf{u}_i$.

Before proceeding, we consider two cases for the number of emitters M. These cases are $M < \min(N, K)$, which corresponds to rank deficiency in the matrix $\mathbf{I}$; and $M \geq \min(N, K)$, which corresponds to the matrix $\mathbf{I}$ being full-ranked. MUSIC, the method that inspired MUSICAL, considered only the rank-deficient case [6], [7], while MUSICAL deals with both the cases. We note that the null space, which is important to both MUSIC and MUSICAL, is easily determinable in the rank deficient case, whereas the full-ranked case does not have a well defined null space. Thus, we consider the two cases separately first and then use a generalized notation that can be used for both the cases while applying MUSICAL.

Case 1: Rank deficient matrix $\mathbf{I}$, $M < \min(N, K)$ — If the matrix $\mathbf{I}$ is rank deficient, then there exists a null space $\mathbf{N} : \{\mathbf{u}_{\sigma_i = 0}\}$ which is orthogonal to $\mathbf{R}$. The physical definition of the range, discussed above, implies that the EMVs are orthogonal to the vectors in $\mathbf{N}$, i.e.

$$\mathbf{G}'(\mathbf{r}'_m) \cdot \mathbf{u}_{\sigma_i = 0} = 0, \tag{12}$$

where $\mathbf{a} \cdot \mathbf{b}$ denotes the vector dot product of the vectors $\mathbf{a}$ and $\mathbf{b}$. Assuming that the fluorophores blink independent of each other, the mapping from $\{\mathbf{G}'(\mathbf{r}'_m); m = 1\,\mathrm{to}\,M\}$ to $\{\mathbf{u}_{\sigma_i \neq 0}\}$ is one-to-one, i.e. each $\{\mathbf{u}_{\sigma_i \neq 0}\}$ can be represented as a unique linear combination of $\{\mathbf{G}'(\mathbf{r}'_m); m = 1\,\mathrm{to}\,M\}$ and vice versa. Consequently, the mapping vectors at other location $\mathbf{r}' \notin \{\mathbf{r}'_m,\ m = 1\,\mathrm{to}\,M\}$ have a non-zero projection on the null space $\mathbf{N}$, i.e.

$$\mathbf{G}'(\mathbf{r}') \cdot \mathbf{u}_{\sigma_i = 0} \neq 0, \text{ if } \mathbf{r}' \notin \{\mathbf{r}'_m,\ m = 1\,\mathrm{to}\,M\}. \tag{13}$$

Thus, an indicator function can be designed which tests the conditions in eqs. (12,13) at different test points $\mathbf{r}'$ in the sample plane.

Case 2: Full-rank matrix $\mathbf{I}$, $M \geq \min(N, K)$ — If $M \geq \min(N, K)$, then the rank of the matrix is $\min(N, K)$, none of the singular values is zero, and consequently a null space does not exist. However, the eigenimages $\mathbf{u}_i$ now correspond to different structural details of the arrangement of the fluorophores and their corresponding σ_i^2 represent the energy (or strength) of the eigenimage $\mathbf{u}_i$. We can choose first few eigenimages with the large eigenvalues as the representative of the structure and assume that the presence of noise may corrupt the details in the eigenimages with smaller eigenvalues. Quantitatively, we can choose a threshold σ_0 such that $\sigma_i < \sigma_0$ is considered small and the corresponding eigenimages can be designated as belonging to the null space $\mathbf{N} : \{\mathbf{u}_{\sigma_i < \sigma_0}\}$ and the space orthogonal to it as the range space $\mathbf{R} : \{\mathbf{u}_{\sigma_i \geq \sigma_0}\}$. We note that such designation of the range and the null space through the threshold σ_0 is not mathematically rigorous but practically useful.
As an example, if the fluorophores are arranged in a line, then the eigenimage with the largest eigenvalue indicates the structure of a line, irrespective of the density of molecules along the line. Similarly with a circle. Thus, the major structure pattern of simple arrangements can still be captured with very few eigenimages corresponding to the large singular values.
Thus, if a point $\mathbf{r}'$ belongs to the structural details characterized by the designated range $\mathbf{R}$, then $\mathbf{G}'(\mathbf{r}')$ for such point has zero projection on the designated null space $\mathbf{N}$, and a non-zero projection otherwise.

Notational generalization of the two cases — The structural arrangement of fluorophores can be represented by the set of eigenimages corresponding to the large singular values, which we loosely call the range and denote as $\mathbf{R}$. The cardinality #$\mathbf{R}$ of $\mathbf{R}$ is defined as the number of eigenimages in $\mathbf{R}$. In the case $M < \min(N, K)$, #$\mathbf{R}$ is equal to M and the range is

rigorously defined. In the case $M \geq \min(N, K)$, #$\mathbf{R}$ is less than $\min(N, K)$ and is user designated. In either case, the null space $\mathbf{N}$ is the space orthogonal to $\mathbf{R}$.

An indicator function can be designed to test if $\mathbf{G}'(\mathbf{r}') \cdot \{\mathbf{u}_i \in \mathbf{N}\} = 0$. If this is so, the vector $\mathbf{G}'(\mathbf{r}')$ belongs to the structure indicated by $\mathbf{R}$. Thus the design goals of MUSICAL are

1) to ensure that #$\mathbf{R}$ can be determined robustly,
2) to design a suitable indicator function which can provide super-resolution, and
3) to mitigate the effect of noise on the indicator function.

Supplementary Note 2. Algorithmic details of MUSICAL

The flowchart of the multiple signal classification algorithm (MUSICAL) is given in Supplementary Figure 1. We discuss each functional block of MUSICAL and its contribution in achieving the goals of MUSICAL.

Sliding window

Instead of considering the whole image stack at once, we consider only small spatial window around a pixel and slide this window across all the pixels. The size of the spatial window is the approximate size of the main lobe of the point spread function (in pixels). Thus, for a PSF resembling an Airy disk, the size of the spatial window can be taken as $N_\mathrm{w} = \mathrm{floor}\left(\frac{1.22\lambda}{\mathrm{NA}w}\right)$. For computational convenience of associating the sliding window with a pixel, we use the closest odd number,

$$N_\mathrm{w} = 1 + 2\,\mathrm{floor}\left(\frac{0.61\lambda}{\mathrm{NA}w}\right). \tag{14}$$

The use of floor (i.e., largest integer smaller than the value) implies that we consider the main lobe to an extent close to but smaller than the span between the zeros.

The use of the sliding window contributes towards the goal 1. Choosing a small spatial window instead of the complete image stack helps because there may be several such windows in which the number of fluorophores is small or zero and thus $\#\mathbf{R}$ is easily determinable. Even for the other windows, unless the number of particles in a window is large and the distribution is random or complicated, few eigenimages can represent the distribution of the fluorophores in the window. Thus, $\#\mathbf{R}$ can be reasonably determined. Thus, the use of window directly helps in ensuring the applicability of the aforementioned core concept for most scenarios.

The use of the sliding window also helps towards goal 3. Since the spatial window spans only the main lobe of the PSF, the noise from the pixels beyond the window does not impact the indicator function computed using this window. Moreover, since the fluorophores in center pixel of this window do not contribute significantly to the pixels beyond the main lobe of the PSF, no significant information is lost in the context of the test points inside the center pixel.

For later reference, the window is slid one pixel at a time and the center pixel of the window is denoted as p. The image stack corresponding to the window centered at p is denoted as $\mathbf{I}_p$. In the context of Supplementary Note 1, $\mathbf{I}$ can be replaced by $\mathbf{I}_p$ and related quantities are calculated with reference to $\mathbf{I}_p$ as the image stack.

Soft window function

Due to the use of sliding window, the indicator function is the most reliable for the test points in the center pixel. For the pixels at the edge of the window, the effect of truncation due to the finite size of the window may be considered abrupt. Thus, we apply a soft-window function, which scales the weights of the pixels according to their proximity to the center pixel. This is elegantly achieved in the mathematical framework of Supplementary Note 1, as discussed next. Let the spatial distribution of the soft window function be given as

$$\mathbf{h} = [h(\mathbf{r}_1) \quad h(\mathbf{r}_2) \quad \ldots \quad h(\mathbf{r}_N)], \tag{15}$$

and the diagonal soft window transformation matrix be given by $\mathbf{H} = \mathrm{diag}(\mathbf{h})$. We obtain the soft windowed image stack $\mathbf{J}_p$ as

$$\mathbf{J}_p = \mathbf{H}\mathbf{I}_p. \tag{16}$$

Further, all mapping vectors are correspondingly transformed as

$$\mathbf{G}''(\mathbf{r}') = \mathbf{H}\mathbf{G}'(\mathbf{r}'). \tag{17}$$

After this step, in the context of Supplementary Note 1, $\mathbf{I}$ and $\mathbf{G}'(\mathbf{r}')$ can be replaced by $\mathbf{J}_p$ and $\mathbf{G}''(\mathbf{r}')$, respectively.

We have used 2-dimensional rotationally symmetric Gaussian function, which is centered at the center pixel $\mathbf{r}_p$ and has a spread (standard deviation) $\rho = w\frac{N_\mathrm{w}-1}{2}$

$$h(\mathbf{r}) = \exp\frac{\|\mathbf{r}-\mathbf{r}_p\|^2}{2\rho^2}. \tag{18}$$

Multiple signal classification

SVD of $\mathbf{J}_p$ yields the eigenimages $\mathbf{u}_{\sigma_i}$ and the corresponding singular values σ_i. In the original form of multiple signal classification (MUSIC), the following indicator function was computed for the test points $\mathbf{r}'_\mathrm{test}$ in the sample plane

$$f(\mathbf{r}'_\mathrm{test}) = \frac{1}{\sqrt{\sum_{\sigma_i<\sigma_0}\|\mathbf{G}'(\mathbf{r}'_\mathrm{test})\cdot\mathbf{u}_i\|^2}}. \tag{19}$$

The indicator function $f(\mathbf{r}'_\mathrm{test})$ demonstrates the following behavior:

$$f(\mathbf{r}'_\mathrm{test}) = \begin{cases} \infty & \text{if } \mathbf{r'}_\mathrm{test} \in \mathbf{L} \\ \text{finite} & \text{otherwise.} \end{cases} \tag{20}$$

where $\mathbf{L}$ represents the fluorophore structures represented by vectors in $\mathbf{R}$. As a consequence, $f(\mathbf{r}'_\mathrm{test})$ is unbounded individually for two closely placed emitters but bounded as test points between them. This allows two closely placed emitters to be

distinguished. We note that even in ideal noiseless measurements, the numerical precision of the computation machine implies that the values are never infinite in practice but limited by the computation precision.

We have modified the indicator function of MUSIC to achieve goal 2, contribute towards goal 3, and enable direct stitching of the MUSIC images of different sliding windows. For the modification, we define the projection distances $d_{\mathrm{PN}}(\mathbf{r}'_{\mathrm{test}})$ and $d_{\mathrm{PR}}(\mathbf{r}'_{\mathrm{test}})$ as follows

$$d_{\mathrm{PN}}(\mathbf{r}'_{\mathrm{test}}) = \sqrt{\sum_{\sigma_i<\sigma_0} \|\mathbf{G}'(\mathbf{r}'_{\mathrm{test}}) \cdot \mathbf{u}_i\|^2}, \tag{21}$$

$$d_{\mathrm{PR}}(\mathbf{r}'_{\mathrm{test}}) = \sqrt{\sum_{\sigma_i\geq\sigma_0} \|\mathbf{G}'(\mathbf{r}'_{\mathrm{test}}) \cdot \mathbf{u}_i\|^2}, \tag{22}$$

where $d_{\mathrm{PN}}(\mathbf{r}'_{\mathrm{test}})$ and $d_{\mathrm{PR}}(\mathbf{r}'_{\mathrm{test}})$ are the projection of $\mathbf{G}'(\mathbf{r}'_{\mathrm{test}})$ on the null space $\mathbf{N}$ and the range $\mathbf{R}$ respectively. Then, we define the indicator function for MUSIC as

$$f(\mathbf{r}'_{\mathrm{test}}) = \left(\frac{d_{\mathrm{PR}}(\mathbf{r}'_{\mathrm{test}})}{d_{\mathrm{PN}}(\mathbf{r}'_{\mathrm{test}})}\right)^{\alpha}, \tag{23}$$

The behavior of the modified indicator function is similar to eq. (20). It has the following additional salient properties

- It incorporates the information of all the eigenimages (both $\mathbf{R}$ and $\mathbf{N}$) whereas the information of $\mathbf{R}$ is completely ignored in eq. (19).
- Through the use of $d_{\mathrm{PR}}(\mathbf{r}'_{\mathrm{test}})$, MUSIC image of each sliding window is automatically scaled with reference to the complete image stack and thus, the MUSIC images positioned in the relevant pixel can simply be added for stitching all the MUSIC images and obtaining the MUSICAL result.
- The value of α, typically chosen more than 1, determines the spread of $f(\mathbf{r}'_{\mathrm{test}})$ for a single fluorophore. With all the parameters remaining the same, increasing the value of α makes the spread narrower and consequently the resolution better. Thus, in the presence of significant amount of noise, resolution of MUSICAL may be improved by simply choosing larger α. On the other hand, it increases the dynamic range of the MUSIC image. Unless mentioned, we have used $\alpha = 4$ for generating the MUSICAL results.
- The modified indicator function is a dimensionless quantity.

Stitching MUSIC images

Theoretically, the test points $\mathbf{r}'_{\mathrm{test}}$ can be any points in the sample region corresponding to the sliding window. But, for the convenience of forming the final image by stitching the results of all the sliding windows, we decimate every pixel into a sub-pixel grid with even number of grid points along each direction and take the centers of the sub-pixels as the test points. Thus, the coordinates of the test points $\mathbf{r}'_{\mathrm{test}}$ in a pixel are given as $(x' + (0.5 + l_x)/L_x, (y' + (0.5 + l_y))/L_y$, where L_x, L_y are the even numbers of sub-pixels per pixel along the x and y directions, l_x, l_y are integers with $|l_x| \leq L_x/2, |l_y| \leq L_y/2$, and x', y' are the conjugate points of the pixel (x, y) in the sample plane.

Suppose we wish to compute the MUSICAL result for the qth pixel. When the sliding windows are centered at the pixels with coordinates

$$x_p = x_q + wn_x; \quad y_p = y_q + wn_y \tag{24}$$

where $|n_x|, |n_y| \leq (N_w - 1)/2$, then qth pixel is a part of these sliding windows and thus MUSIC's indicator function is computed for the test points in the qth pixel. Let us denote the indicator function computed for a sliding window with center pixel (x_p, y_p) by $f_{x_p,y_p}(\mathbf{r}'_{\mathrm{test}})$. Then, the MUSICAL result at $\mathbf{r}'_{\mathrm{test}}$ in the qth pixel can be obtained by

$$F\left(\mathbf{r}'_{\mathrm{test}} \in q\mathrm{th\ pixel}\right) = \sum_{n_x}\sum_{n_y} f_{x_p,y_p}\left(\mathbf{r}'_{\mathrm{test}}\right) \tag{25}$$

Supplementary Note 3. Concept of range and null space of matrix and relationship to singular value decomposition

Any matrix $\mathbf{A}$ of size $m \times n$ can be interpreted as a linear mapping from an $n-$dimensional input space to an $m-$dimensional output space. Depending on the nature of $\mathbf{A}$, although the output space is $m-$ dimensional, the outputs may be restricted to a smaller subspace of this $m-$ dimensional space. Such nature of $\mathbf{A}$ is characterized by rank$(\mathbf{A}) < \mathbf{m}$. The actual output subspace of the matrix $\mathbf{A}$ is called the range $\mathbf{R}$ of the matrix $\mathbf{A}$ and the matrix $\mathbf{A}$ is said to span the range $\mathbf{R}$. The complementary subspace to the range $\mathbf{R}$, which is not spanned by the matrix $\mathbf{A}$ is called the null space $\mathbf{N}$. Consider the following matrix as an example:

$$\mathbf{A} = \begin{bmatrix} 1 & 0 & 2 & 0 \\ 0 & 1 & 0 & 2 \\ 1 & 1 & 2 & 2 \end{bmatrix} \tag{26}$$

It is evident that although the above matrix $\mathbf{A}$ maps inputs to a $3-$dimensional space, the mapped outputs lie on a $2-$dimensional plane only. The rank of this matrix $\mathbf{A}$ is 2 and the aforementioned plane is the range of $\mathbf{A}$. All the points in the $3-$dimensional space except the this plane belong to the null space. However, for the matrix $\mathbf{A}$ below, the null space is empty:

$$\mathbf{A} = \begin{bmatrix} 1 & 0 & 2 & 0 \\ 0 & 1 & 0 & 2 \\ 1 & 1 & 1 & 1 \end{bmatrix} \tag{27}$$

In the situation that the input space is smaller than the output space, i.e. $m > n$, the null space is definitely not empty. For example, if points on a line (a one dimensional input space) are linearly mapped to a two-dimensional or three-dimensional output space, the mapped points would still be confined to a line.

Since the range of a matrix $\mathbf{A}$ is spanned by the linear mapping $\mathbf{A}$, any point in the range can be expressed as a linear combination of columns in $\mathbf{A}$. On the other hand, any point in the null space cannot be expressed as a linear combination of the columns of $\mathbf{A}$. This implies that the entire null space is orthogonal to the columns of $\mathbf{A}$ as well as to the range of $\mathbf{A}$.

Any point in an $n-$ dimensional space can be represented as a linear combination of n independent vectors. Such a set of n independent vectors is called the set of basis vectors or simply the basis vectors. Although there are infinitely many such sets, it is often desirable to choose basis vectors with specific properties, such as the Euclidean norm of each vector being one, or the basis vectors being orthogonal to each other, or one of the basis vectors being along the direction of maximum variance for a cluster of measured points. Since MUSICAL exploits the range and the null space and their mutual orthogonality, it is desirable to choose a set of basis vectors which allows for convenient identification of the range and the null space, as well as inherently uses orthogonality. Singular value decomposition fulfils these requirement easily. This is because the singular vectors are mutually orthogonal, form a set of basis vectors, and satisfy the following: $\mathbf{A}\bar{\mathbf{v}}_\mathbf{i} = \sigma_\mathbf{i}\bar{\mathbf{u}}_\mathbf{i}$, where $\bar{u}_i$, $\bar{v}_i$, and σ_i are the ith left singular vector, right singular vector, and singular value of the matrix $\mathbf{A}$, respectively. If $\sigma_i \neq 0$, it implies that $\bar{u}_i$ can be represented as a linear combination of the columns in $\mathbf{A}$, and thus $\bar{u}_i$ lies in the range. Similarly, if $\sigma_i = 0$, $\bar{u}_i$ cannot be represented as a linear combination of the columns in $\mathbf{A}$, and thus belongs to the null space.

Supplementary Note 4. The choice of σ_0 and the effect of noise

The presence of noise affects both the range and the null spaces of the measurement matrix $\mathbf{I}$ (or the sliding window $\mathbf{J}_p$). As a result of noise, not only the singular values σ_i, but the eigenimages $\bar{u}_i$ also change to a certain extent. The most direct effect of noise is that the singular values may no longer be zero even in the rank-deficient case. Yet, the null space can be determined by suitable selection of the threshold σ_0. The indirect impact of noise is that $d_{\mathrm{PN}}(\vec{r}'_{\mathrm{test}}) \neq 0$ for $\vec{r}'_{\mathrm{test}} \in \mathbf{R}$. Thus, the indicator function $f(\vec{r}_{\mathrm{test}})$ does not have a clear classification as indicated in eq. (20). It rather has a continuous spread as we move away from a point $\vec{r}'_{\mathrm{test}} \in \mathbf{R}$. In this situation, higher value of α helps in reducing the spread.

We provide two rules of thumb to select the value of the threshold σ_0. If the signal to noise ratio SNR = $\|\mathbf{I}\|/\|\mathbf{N}\|$ is known, then σ_0 is chosen as

$$\sigma_{\max}/\sigma_0 = \mathrm{SNR} \tag{28}$$

where $\sigma_{\max}$ is the maximum singular value observed for the singular values computed for all the sliding windows. If the SNR is not known, then the value of σ_0 is chosen to be slightly less than the value where a knee feature is observed in the logarithmic plot of singular values of all the sliding windows. Here, knee feature is characterized by a point before which singular values show a fast decaying characteristics and after which the singular values decay slowly.

We consider SynEx1 for the case $M < \min(N, K)$. It is seen in Supplementary Figure 3(a) that in the absence of noise, only four singular values are non-zero while the remaining singular values drop to zero or numerical noise of the computer. They are not shown for convenience of visualization. Thus, the null space is clearly defined in this case. The modified indicator function with $\alpha = 1$ computed for a grid of test points is plotted in Supplementary Figure 3(b) and the zoom-in of the center pixel is shown in Supplementary Figure 3(c). The result clearly shows high values of the indicator function at the test points closest to the emitter locations. Lastly, the use of $\alpha = 4$ further reduces the background, as seen in Supplementary Figure 3(d).

Next, we consider SynEx2 for the case $M \geq \min(N, K)$. We consider the central region of the distribution, i.e. the sliding window for the center most pixel in the image stack. This window of 7×7 pixels has 108 emitters. The singular values for this window are shown in Supplementary Figure 4. It is seen that none of the singular values are zero, indicating that the null space is not rigorously defined. However, the decay of singular values indicate that we may choose σ_0 to allow different definitions of the null space in the computation of the modified indicator function. For a high value of σ_0, the structure shown in bottom-left of Supplementary Figure 4 can be identified using the modified indicator function. For subsequently smaller value of σ_0, the modified indicator function shows increasingly more details.

Lastly, we consider the presence of noise in SynEx1. In the presence of noise, all the singular values are non-zero, as seen in Supplementary Figure 3(a)). Thus, the null space cannot be clearly determined. Nevertheless, a knee can be observed after 4 singular values (indicated using gray dashed lines in Supplementary Figure 3(a)). Thus, using $\log_{10} \sigma_0 = -1.2$, where the knee is approximately noted, the modified indicator functions are plotted using $\alpha = 1$ in Supplementary Figure 3(e,f) and using $\alpha = 4$ in Supplementary Figure 3(g). The indicator function shows a continuous spread in the presence of noise and the spread is significantly smaller for $\alpha = 4$.

Supplementary Note 5. Example of eigenimages

We illustrate eigenimages and the corresponding eigenvalues for SynEx1 in Supplementary Figure 6. Only the first six eigenimages are used for convenience. It is seen that the eigenimages with non-zero eigenvalues represent information about the structure and the eigenimages with zero eigenvalues represent noise patterns. Other eigenimages also correspond to zero eigenvalues and represent random noise patterns. If the emitters were not blinking, all the images in the image stack would be the same and thus only one eigenvalue would be non-zero and would represent simply the image repeated in the entire image stack. On the other hand, if the emitters would be emitting in synchronism with each other, the image stack could be represented by only two images, one in which all the emitters are emitting and the other being the dark image. Even in this case, only one eigenvalue would be non-zero and the corresponding eigenimage would be the image corresponding to emissions from the emitters. It is the phenomenon of blinking that introduces diversity in the images and more than one non-zero eigenvalues, each of which represents a different pattern from the image stack.

Supplementary Figure 6 also presents the projections of the PSFs of test points on the eigenimages. It is seen that the projection at the actual emitter locations is quite high for the first eigenimage. In other eigenimages with non-zero eigenvalues, the projections at the actual emitter locations are smaller than the projection for the first eigenimage but not equal to zero. The projections at the actual emitter locations on the fifth and sixth eigenimages are zero. These properties are more obvious in the log scale of the projections shown in the bottom row of Supplementary Figure 6. The projections of the PSF are similarly zero at the emitter locations for all the eigenimages with zero eigenvalues. At other locations, the projections on the eigenimages with zero eigenvalues is not consistently zero. These properties of the projections on the eigenimages in the null space are the critical feature of MUSIC and MUSICAL and are exploited in their indicator functions.

Supplementary Note 6. Discussion on the role of the parameter α

Here, we discuss further on the role of the parameter α in MUSICAL. Irrespective of the definition of the indicator function and the value of α, resolution between two features is theoretically achieved in the case of noise-free measurements if the indicator function is finite at a test point between the features because the contrast is perfect according to eq. (20). However, practically the indicator function is not infinite at the location of the emitters. Further, it is not necessary that the actual emitter location is also a test point. Nevertheless, a dip in the value of the indicator at a point between two features indicates non-zero contrast and thus computationally resolved features. However, for visual resolution, sufficient contrast is essential. Since the indicator function of MUSICAL is inherently non-linear through the presence of d_{PN} in the denominator, α only tweaks the amount of non-linearity, consequently influencing three aspects of MUSICAL result. The first consequence is obvious; α obviously controls the contrast of the MUSICAL image. The second consequence is somewhat subtle; the value of α determines the order of non-linearity in the stitching of the MUSICAL results of sliding windows through eq. (25). The higher the order, lower is the contribution of the off-center sliding windows to the final MUSICAL image. A small value of α, such as 1 or 2, implies larger contribution from the off-center sliding windows which are more susceptible to noise. A large value of α implies less contribution from the off-center sliding windows, which do contain some information about the test points. The third consequence is that a high value of α non-linearly scales the less prominent features and makes them visually less apparent. The features may be less prominent due to lower number of emissions or less fluctuations. On the other hand, small value of α emphasises the non-zero and non-uniform background, such as expected in the MUSICAL indicator function due to its characteristic in eq. (20). We heuristically found that $\alpha = 4$ provides a good trade-off for all the three aspects in general.

Supplementary Note 7. Results of in-vitro sample 3 for different excitation powers

We considered another in-vitro sample, Sample 3, for which image stacks were acquired at excitation laser powers 0.93 W cm^{-2}, 10.3 W cm^{-2}, and 205.6 W cm^{-2}. The results are shown in Supplementary Figure 13. The MUSICAL results show sharper images than the mean images for all the three powers, see Supplementary Figure 13(c,f,i). Comparison of MUSICAL

results at the cross-sections D1-D3 are shown in Supplementary Figure 13(j). It is notable that as the power decreases from 205.6 W cm^{-2} (D3) to 10.3 W cm^{-2} (D2), FWHM deteriorates from 38.1 nm to 248.4 nm. However, as the power decreases from 10.3 W cm^{-2} to 0.93 W cm^{-2}, the FWHM deteriorates by only 20 nm. The FWHM of mean intensities at the cross-sections D1 and D2 are 320 nm and 336 nm, respectively. Thus, MUSICAL result is slightly better than the unprocessed image stack even when the excitation power is low.

Thus, collecting the results shown for in-vitro sample 2 in Fig. 2 and the result for sample 3, we form an empirical plot of power response of MUSICAL shown in Supplementary Figure 13(k). We note that there are three factors responsible for the observed steep curve in Supplementary Figure 13(k). As shown in Dempsey et. al [8], reduction of excitation laser intensity has two effects that affect the captured signal directly. First, reduced intensity reduces the number of photons emitted per switching cycle, thus deteriorating the SNR as well as the SBR of the image stack. Notably the SBR does not change significantly in the flat regions of the curve in Supplementary Figure 13(k). Second, reduced excitation intensity reduces the off-switching rates and thus increases the duty cycles, which translates to less dynamic variation in blinking or flatter statistics. Further, a third effect is the survival rate of emitters, which reduces with increasing excitation power. We believe that these three effects compete with each other to result into the empirical plot of Supplementary Figure 13(k). We note here that since the total acquisition time duration is significantly shorter than the time needed to reach the equilibrium on-off duty cycle (400-600 seconds [8]), the duty cycle does not reach the equilibrium value, which is almost the same for all the power values.

Supplementary Note 8. MUSICAL and sensitivity to the camera noise

MUSICAL result for in-vitro sample 3 provides interesting observation for low powers. Supplementary Figure 13(b,e) show some small particle-like artifacts, with relatively less intensity than the main structures. We discuss the cause of the appearance of these artifacts. For this, we fuse the MUSICAL images corresponding to image stacks captured with 0.93 W cm^{-2} and 10.3 W cm^{-2} and show the fused result in Supplementary Figure 15. Supplementary Figure 15(a) shows the fused images where the MUSICAL results have been shifted to compensate for the sample drift between the two acquisitions, whereas Supplementary Figure 15(b) shows the fusion without compensating for the sample drift. We highlight, using white arrows, some of the artifact in the fused images. Notably in Supplementary Figure 15(b), the artifacts are at the same location but the samples are not aligned, as seen using two distinct non-overlapping red and green casts in the sample details. On the other hand, Supplementary Figure 15(a) shows the samples aligned but one red and one green artifact for each artifact shown in Supplementary Figure 15(b). In other words, if the samples are aligned the artifacts do not overlap and if the artifacts are aligned, the samples are shifted.

This indicates that the artifacts are neither sample related nor algorithm induced, because the particles would have moved with the sample in either of these cases. Interestingly, the standard deviation maps in Supplementary Figure 15(c,d) of the image stacks show that some background pixels, where the artifacts were observed have larger standard deviations than the other background pixels. Upon further analysis, we found that the dark noise characteristics of the pixels of the sCMOS camera used for measurements are not uniform despite the in-built hot-pixel correction. Particularly, some pixels exhibit significantly larger standard deviations than the rest as seen for a randomly selected region in Supplementary Figure 15(f), although the mean values appear relatively uniform as seen in Supplementary Figure 15(e).

We used the semi-synthetic example SynEx4Bck, which is a fusion of synthetic data and measured dark noise, see eq. (32) in Supplementary Methods, to validate the occurrence of such artifacts. The mean and standard deviations of the image stack are shown in Supplementary Figure 16(a,b) and demonstrate roughly similar behaviour as the mean and standard deviation maps of Sample 3. The SBR is about 1.5, as noted from the mean image in Supplementary Figure 16(a). The MUSICAL result in Supplementary Figure 16(c) shows particle-like artifacts in the background in addition to the pronounced effect of side lobes due to poor SBR. We note that the maximum MUSICAL intensity for SynEx4Bck is about 72. Thus, according to Supplementary Figure 14(e), side lobes with intensity of about 6.5 are expected, which agrees with the observed values in Supplementary Figure 16(c).

Supplementary Note 9. Compensating for known noise pattern of the camera and suppressing side lobes

We demonstrate here a method each for compensating for known camera noise pattern and for suppressing side lobes. While these approaches are not needed when the SBR is sufficiently high, they may be useful for low SBR.

If an image stack of the dark noise of the same image region can be captured, its singular value decomposition can be used to suppress these artifacts provided that they are not too close to the the features of interest. Suppose the sliding window at a pixel p gives the small image stack $\mathbf{I}_p$ and noise stack $\mathbf{N}_p$ and their respective eigenimages are denoted as $\mathbf{u}_i^{\mathrm{meas}}$ and $\mathbf{u}_i^{\mathrm{noise}}$, then the net projection on the dark noise space onto the measurement space is given as

$$d = \frac{\sum_i \sum_j \mathbf{u}_i^{\mathrm{meas}} \cdot \mathbf{u}_j^{\mathrm{noise}}}{N^{\mathrm{meas}} N^{\mathrm{noise}}} \tag{29}$$

where N^{meas} and N^{noise} are the number of eigenimages of $\mathbf{I}_p$ and $\mathbf{N}_p$, respectively. Then, a scale factor $\mathrm{scale}(p)$

$$\mathrm{scale}(p) = 1 - |d| \tag{30}$$

can be used to scale the indicator function of MUSICAL. The scale factor for SynEx4Bck is shown in Supplementary Figure 16(e).

For compensating for the side lobes, we use non-linear weighing of the MUSICAL indicator function, where the value of the indicator function at each test point $\mathbf{r}_{\text{test}}$ is weighed by $w(\mathbf{r}_{\text{test}})$, a sigmoid function defined as follows

$$w(\mathbf{r}_{\text{test}}) = \frac{1}{1 + \exp\left(-\beta\big(f(\mathbf{r}_{\text{test}}) - f_0\big)\right)} \tag{31}$$

where β is a control parameter that determines the rate at which the function decays. Here, we choose $\beta = 0.25$. Further, f_0 is the soft threshold for the intensity. We recommend f_0 to be a fraction (we have used 0.3) of the maximum intensity of the MUSICAL result. For example, here we have used $I_0 = 20$ for SynEx4Bck, which is about 0.3 times the maximum MUSICAL intensity. The weights thus calculated for SynEx4Bck are plotted in Supplementary Figure 16(f). As seen in Supplementary Figure 16(f), the indicator function does not change much if its value is more than 40.

For SynEx4Bck, the MUSICAL result after incorporating both $\text{scale}(p)$ and $w(\mathbf{r}_{\text{test}})$ is shown in Supplementary Figure 16(d). Both the artifacts and the side lobes are significantly suppressed in this result as compared to the original MUSICAL image shown in Supplementary Figure 16(c).

As a further example, we show the effect of $w(\mathbf{r}_{\text{test}})$ on the MUSICAL image of sample 2 in Fig. 2(m) of the main paper. We use $\beta = 1$ and $f_0 = 6.85$ which is approximately 0.3 times the maximum MUSICAL intensity. The results are shown in Supplementary Figure 17. The MUSICAL image with $w(\mathbf{r}_{\text{test}})$ has a significantly lower background as evident in both Supplementary Figure 17(b,c).

Supplementary Note 10. Comparison between MUSICAL and SMLM techniques

The intensity plots of STORM and MUSICAL results at the cross-sections B1-B4 of region B in in-vitro sample 1 shown in Fig. 1(f,g) are given in Supplementary Figure 18(a,b). The intensity plots of STORM and MUSICAL at the cross-sections C2-C3 of region C of sample 1 shown in Fig. 1(i,j) are given in Supplementary Figure 18(c,d).

The cross-sections B1-B4 represent a situation of branching of a filament into two, where B1 shows clearly separated branches, B2 shows a line very close to the junction, B3 is approximately on the junction, and B4 is the unbranched filament. In Supplementary Figure 18(a,b), the STORM and MUSICAL results at sections B1 and B4 point to similar inferences, i.e. two clearly separated branches for B1 and an unbranched filament for B4. However, MUSICAL and STORM results at B2 show quite different results. STORM shows approximately 5 peaks at about 193 nm, 233 nm, 320 nm, 380 nm, and 493 nm, while MUSICAL shows three peaks at 161 nm, 228 nm, and 309 nm. It is indeed difficult to conclude the structure at B2. The results of MUSICAL and STORM for B3 are also interesting. MUSICAL shows two separable but closely located maxima (separated by about 33 nm) with the minimum between them located at about 250 nm. On the other hand, STORM shows a non-smooth plateau spread from about 220 nm to 320 nm.

STORM and MUSICAL results for region C are shown in Fig. 1(i,j). MUSICAL clearly indicates a branching but STORM does not. In the cross-section plots for C2 (just before branching, Supplementary Figure 18(c)) and C3 (after branching, Supplementary Figure 18(d)), it is seen that STORM gives only one maximum while MUSICAL gives two maxima for C3. Also, the maximum of STORM for C3 is closer to the right side maximum generated by MUSICAL.

For further analysis, we use synthetic examples SynFork1 and SynFork2 to emulate forks such as seen in regions B and C of sample 1. SynFork1 and SynFork2 have similar geometry but different blinking rates. Details about these appear in Supplementary Methods. It suffices to mention here that SynFork2 has significantly slow blinking than SynFork1. The mean images of the image stack, STORM result, and MUSICAL result are respectively shown in Supplementary Figure 19(a-c) for SynFork1 and Supplementary Figure 19(d-f) for SynFork2. It is seen that STORM can reconstruct the fork with good fidelity only when the blinking is extremely sparse. The nature of STORM result for SynFork1 with speckles between the prongs matches with the STORM result for the fork in Fig. 1(f).

To illustrate the effect of presence of emitters placed closely in a small volume on STORM and MUSICAL, we consider synthetic example SynSTORM, the details of which are provided in Supplementary Methods and the layout is shown in Supplementary Figure 20(a,f). The width of each of the five lines is about 7 nm such that each line mimics an actin filament. The two closest and parallel lines are separated by 50 nm. The top row corresponds to short dark states ($\tau_{\text{on}} = 0.05$s, $\tau_{\text{off}} = 50$s) while the bottom row corresponds to long dark states ($\tau_{\text{on}} = 0.05$s, $\tau_{\text{off}} = 250$s), where τ_{on} and τ_{off} represent average time spent by molecules in the bright (on) and dark (off) states respectively.

In addition to the imaging result of STORM shown in Supplementary Figure 20(d,i), we also provide localization results of STORM in Supplementary Figure 20(e,j). In the case of long dark states, the number of bright fluorophores at a given time is small even at the junction. Thus, in comparison to MUSICAL result (Supplementary Figure 20(h)), the performance of STORM (Supplementary Figure 20(i)) is superior in this case. On the other hand, in the case of shorter dark states, the number of bright fluorophores in a small volume along the parallel lines is sufficiently high to render STORM ineffective in resolving the lines (Supplementary Figure 20(d)). MUSICAL can, however, still resolve the lines. Further, the MUSICAL result and the details reconstructed at the lines and junction are similar in both the cases.

Supplementary Note 11. Comparison of the computational aspects of super-resolution techniques

We present a succinct juxtaposition of the computational aspects of MUSICAL with the other methods in Supplementary Table 1. While the number of frames required by MUSICAL is very small, SOFI and the single iteration ESI have similarly low requirement; the basic condition for all three is capturing a statistically significant sample of blinking. On the other hand, MUSICAL allows any scale of sub-pixelation without additional iterations unlike those in ESI or the cross-correlations in SOFI. MUSICAL scores better than 3B in computation time but 3B outperforms MUSICAL in image sharpness as the signal to noise ratio becomes poorer, due to its iterative rejection of less likely localization candidates. Further, as compared to SOFI, ESI, as well as 3B, MUSICAL requires the PSF to be known with sufficient accuracy although its sensitivity to the error in PSF estimation is reduced by choosing a sufficiently high value of α. The only aspect where MUSICAL is somewhat heuristic is in the choice of its control parameter σ_0. However, the rules of thumb noted in Supplementary Note 4 are sufficient in most cases. Further, the appearance of grid artifacts despite using the modified indicator function and $\alpha \geq 2$ is a good indication of an inappropriate choice of the value of σ_0.

MUSICAL and Spatial Covariance Reconstructive (SCORE) Super-Resolution Fluorescence Microscopy

Spatial Covariance Reconstructive (SCORE) Super-Resolution Fluorescence Microscopy [9] is a recent method that also uses eigenimages, though quite differently from MUSICAL. It provides resolution comparable to SOFI and ESI. Here, we highlight some important conceptual differences between SCORE and MUSICAL:

- Score uses only those eigenimages that belong to the range of the measurement matrix. On the other hand, the use of eigenimage in the null space is the core of MUSICAL and is even more important than the use of eigenimages in the range.
- MUSICAL uses the information of PSF not only for computing the projections on the range and the null space but also to incorporate sliding window function, which is critical in reducing the impact of noise and other emitters distant from the emitters in the sliding window. SCORE does not use any sliding window; its use of PSF is limited to the computation of the projections onto the eigenimages in the range.
- SCORE casts the reconstruction as a minimization problem of minimizing the difference between the measured and estimated covariances after estimating an initial covariance matrix using an exponential indicator function of the projection. MUSICAL does not need to solve an iterative optimization and is expected to be more computation efficient for this reason.

SUPPLEMENTARY METHODS.

Implementation and values of control parameters of MUSICAL and other methods

Here, we discuss the details of the implementations and values of control parameters for all the methods used in this paper for reporting results.

MUSICAL — MUSICAL has been implemented on Matlab 2012b. Parallelization has not been implemented. For generating the results in the papers, camera's offset value of 80 is subtracted for experimental images and synthetic examples for which the signal to background ratio is defined. Gaussian soft window of size 7×7 pixels is used. The standard deviation of the Gaussian soft window is 3 pixels. $\alpha = 4$ is used. The signal to background ratio of the mean image after subtraction of the offset was used as the SNR estimate for selecting σ_0 as discussed in Supplementary Note 4. If the result appeared unsatisfactory, the knee criterion was used for generating the MUSICAL result. For synthetic examples without noise, the knee criterion is used. The point spread function used in MUSICAL is assumed to be the standard Airy disk computed using the specified emission wavelength, numerical aperture, magnification, and pixel size.

STORM [10] — The rainSTORM Matlab code provided by the authors of [11] was used for generating the STORM images. Least squares based fitting of Gaussian function along x and y axes is done independently on the pixels which show non-zero difference from all immediate neighbors. We chose a region of interest of 3 pixels, which translates to using 7 pixels along either direction for fitting Gaussian function. We tried values 4 and 5 for region of interest as well, but found that using 3 as region of interest gave sharper images. Further, we chose an estimate of the standard deviation of the Gaussian function as 2.9 pixels, which corresponds to 188.5 nm. However, as noted in the user guide, the choice of this parameters is almost inconsequential since the range of standard deviation of Gaussian function is quite large. The tolerance is set to 0.2, as recommended in the user guide. The number of iterations for fitting the Gaussian function is set to 10, which is more than the recommended value 6. For Fig. 3(b) of the main paper, the STORM and PALM results provided with the dataset are used. Suitable colorbar has been used for better and brighter visualization of these results.

DeconSTORM [12] — Matlab implementation of deconSTROM provided by the authors of [12] on the project webpage [13] is used. Default values were used for all the control parameters except the parameter β, i.e. the probability of an inactive emitter will become active in the next frame. Relevant optical parameters of the experiment were provided. Different values in the range $\beta \in [6.5 \times 10^{-5}, 0.65]$ were attempted and the best result was reported. $\beta \sim 6.5 \times 10^{-5}$ suited the tubulin long sequence data in Data-SMLM because of very sparse blinking. $\beta \sim 0.65$ suited the tubulin high density data in Data-SMLM and SynEx2 due to non-sparse blinking. $\beta \sim 0.0065$ was used for the remaining data. Sub-pixelation by a factor of 8 was used.

3B [14] — The 32-bit ImageJ plugin for 3B provided by the authors of [14] on their project page [3] was used for 3B computation. The default hardcoded values of the control parameters were used. Relevant optical parameters of the experiment were provided. For generating the 3B image using the localization results of 3B, reconstructed pixel size of 3.25 nm (sup-pixelation by a factor of 20) and reconstruction blur PSF of 30 nm was used. Results in Fig. 4(a,b) and Supplementary Figure 21 are obtained after 50 iterations and results in Fig. 4(c,d) of the main paper are obtained after 65 iterations.

ESI [15] — Fiji plugin provided by the authors of [15] was used for ESI computation. For the results generated in Fig. 4 of the main paper, two iterations of order 2 each were run. The number of images binned in the first iteration was a factor of the number of frames K closest to the $\sqrt{K}$. Number of bins for entropy was set as 100. Only 1 iteration of ESI was used to generate the result. Logarithmic scale was used for Fig. 4(c,d) of the main paper.

SOFI [16] — Matlab code provided by Dr. Joerg Enderlein for the article [16] was used for SOFI. Cumulants of order 5 were used for Fig. 4(a,b) and Supplementary Figure 21 and order 6 were used for Fig. 4(c,d) of the main paper. Sub-pixelation by a factor of 5 was used.

Color scale used for all super-resolution methods — The non-linearity of all the super-resolution methods mandates the use of a heuristic range of intensities for better visualization of the results. We have clipped the maximum values of the intensity in the following manner. For a result, the cumulative histogram is computed using 10000 uniformly sized bins. The bin at which the cumulative histogram reaches 99.9% is identified. The colorbar of the result is clipped at the center of this bin. Thus, only 0.1% pixels in the result have intensity higher than the maximum intensity of the colorbar used for visualization. For MUSICAL, typically 99.96% is used instead of 99.9%.

Machine used for computation — The results of all the methods were generated using a Dell Precision T5610 workstation with 64-bit Intel Xeon CPU E5-2630 v2 2.60 GHz and 64 GB RAM. The 32-bit ImageJ plugin of 3B [14] was used, however on the same system. The multicore ImageJ implementation of ESI [15] was used. No parallelization was incorporated for SOFI, deconSTORM, and MUSICAL.

Synthetic examples

SynEx1: This example consists of four emitters arranged at the corners of a square of size 30 nm whose corners lie on the x and y axes. For this example, we assume that the image is captured on 7×7 pixel array only, which corresponds to N_w in eq. (14). Its image stack contains $K = 49$ frames. Since the pixel array is of size 7×7 only, there is only one sliding window. This example is used to illustrate the effect of noise and the value of α on the modified indicator function.

SynEx2: This example consists of four circles, each of radius 500 nm. Each circle has 180 emitters uniformly located along the circumference of the circle. The centers of the circles are located at the four corners of a square such that the distance between the diagonal corners is 1100 nm and the corners lie on the axes. All the other parameters of the measurement system are the same as SynEx1, except that each image in the temporal image stack is of size 51×51 pixels. This example is used to illustrate MUSICAL imaging and the effect of the threshold σ_0 for the case $M \geq \min(N, K)$.

SynEx3: SynEx3 has 500 emitters arranged along a line of 200 nm. The image stack has $K = 1000$ frames. It is used to quantify full width at half maximum (FWHM) and side lobe level as a function of signal to background ratio.

SynEx4: SynEx4 has exactly the same details as SynEx2, except that the image stack of SynEx4 has 1000 frames. Then, an image stack, BckEx4, captured for a random region in the actual sCMOS sensor without any laser excitation is fused with the SynEx4 to form a new image stack SynEx4Bck as below

$$\mathrm{SynEx4Bck}(p,k) = 100\frac{\mathrm{SynEx4}(p,k)}{\max\limits_{p',k'} \mathrm{SynEx4}(p',k')} + \mathrm{BckEx4}(p,k) \tag{32}$$

where p, k denote a pixel and a frame, respectively. Here, 100 is used since the average intensity value of the image stack BckEx4 is 100. Thus, SynEx4Bck has SBR 2. Only SynEx4Bck is used. Results for this example appear in Supplementary Note 8.

SynPairDelX: This set of synthetic examples consist of a sample containing two lines of 500 nm each. Each line contains 51 emitters placed uniformly at a separation of 10 nm. The distance between the two lines is characterized by Δx, as shown in Fig. 1(e) of the main paper. One image stack of 1000 frames is generated for each value of Δx, which varies between 20 nm and 60 nm. The file with name SynPairDelX500Angstrom represents the image stack with $\Delta x = 500$ Å$= 50\mathrm{nm}$, i.e. . The image stacks are noise free.

SynPeriod: SynPeriod which has 33 emitters placed along 3 lines which are 100 nm apart. Each line has 11 emitters uniformly placed along a length of 500 nm, consecutive emitters being 50 nm apart. Example contains 1,000 frames captured at a frame rate of 200 frames per second and the SBR is 100.

SynFork1 and SynFork2: SynFork1 and SynFork2 have a geometry similar to the fork seen in Fig. 1(b3) of the main paper. In each of them, the fork has two prongs with an angle of 30° between them attached to a stem; each prong and the stem are 500 nm long and have uniformly distributed 200 emitters. Image stack contains $K = 1000$ frames and has SBR 10.

SynSTORM: Image stacks for the SynSTORM are simulated using Matlab code testSTORM, provided by the authors of [11]. Photon emission rate of 100,000 photons sec^{-1}, average time of 50,000 seconds before bleaching occurs, and emission wavelength of 510 nm are used. The parameters of the imaging system match the experimental system. No background is assumed (background set to 0). The image stacks contain 3000 frames each, taken at 20 frames per second.

Simulation of blinking: SynPairDelX image stacks, SynEx1, SynEx2, SynEx3, SynEx4, SynPeriod, SynFork1 and SynFork2 are generated assuming Poisson blinking model, where the length of switching times t_{on} and t_{off} are computed using a Poisson distribution with the average on time τ_{on} and the average off time τ_{off}, respectively. We have used $\tau_{\mathrm{on}} = 0.3\ \mathrm{ms}, \tau_{\mathrm{off}} = 0.7\ \mathrm{ms}$ for SynEx1 and SynPeriod; $\tau_{\mathrm{on}} = 3\ \mathrm{ms}, \tau_{\mathrm{off}} = 7\ \mathrm{ms}$ for SynEx2, SynEx3, and SynEx4; $\tau_{\mathrm{on}} = 2\ \mathrm{ms}, \tau_{\mathrm{off}} = 998\ \mathrm{ms}$ for SynFork1; $\tau_{\mathrm{on}} = 0.2\ \mathrm{ms}, \tau_{\mathrm{off}} = 999.8\ \mathrm{ms}$ for SynFork2; and $\tau_{\mathrm{on}} = 0.3\ \mathrm{ms}, \tau_{\mathrm{off}} = 999.7\ \mathrm{ms}$ for SynPair stacks. The photon emission rate is assumed to be 100,000 photons sec^{-1}.

Simulation of images: All the examples assume an emission wavelength $\lambda = 510\mathrm{nm}$, a TIRF system of NA =1.49, magnification 100X, and sCMOS camera with pixel size 65 nm.

Simulation of noise: For the example SynEx2 used in Supplementary Note 11, we add camera's offset value of 100 and add Poisson noise. In order to achieve a desired SBR, we scale the intensities in SynEx2 between 0 and 100(SBR-1) and add the imaging system's offset value of 100 to the scaled intensities. Then Poisson noise is added to each intensity value. Similarly, the offset and Poisson noise is added to SynEx3. Offset value of 100, SBR value of 10, and Poisson noise is used for SynFork1 and SynFork2.

Supplementary References

[1] Born, M. & Wolf, E. *Principles of optics: electromagnetic theory of propagation, interference and diffraction of light* (Cambridge university press, 1999).

[2] Hou, S., Solna, K. & Zhao, H. A direct imaging algorithm for extended targets. *Inverse Problems* **22**, 1151 (2006).

[3] Cox, S. 3b microscopy analysis software (2013). URL `http://www.coxphysics.com/3b/#download`.

[4] Bovellan, M. *et al.* Cellular control of cortical actin nucleation. *Current Biology* **24**, 1628–1635 (2014).

[5] Charras, G. T., Hu, C.-K., Coughlin, M. & Mitchison, T. J. Reassembly of contractile actin cortex in cell blebs. *The Journal of cell biology* **175**, 477–490 (2006).

[6] Chen, X. & Agarwal, K. MUSIC algorithm for two-dimensional inverse problems with special characteristics of cylinders. *IEEE Transactions on Antennas and Propagation* **56**, 1808–1812 (2008).

[7] Agarwal, K. & Chen, X. Applicability of MUSIC-type imaging in two-dimensional electromagnetic inverse problems. *IEEE Transactions on Antennas and Propagation* **56**, 3217–3223 (2008).

[8] Dempsey, G. T., Vaughan, J. C., Chen, K. H., Bates, M. & Zhuang, X. Evaluation of fluorophores for optimal performance in localization-based super-resolution imaging. *Nature Methods* **8**, 1027–1036 (2011).

[9] Deng, Y., Sun, M., Lin, P.-H., Ma, J. & Shaevitz, J. W. Spatial covariance reconstructive (score) super-resolution fluorescence microscopy. *PloS one* **9**, e94807 (2014).

[10] Rust, M. J., Bates, M. & Zhuang, X. Sub-diffraction-limit imaging by stochastic optical reconstruction microscopy (STORM). *Nature Methods* **3**, 793–796 (2006).

[11] Rees, E. J. *et al.* Blind assessment of localisation microscope image resolution. *Optical Nanoscopy* **1**, 1–10 (2012).

[12] Mukamel, E. A., Babcock, H. & Zhuang, X. Statistical deconvolution for superresolution fluorescence microscopy. *Biophysical journal* **102**, 2391–2400 (2012).

[13] Zhuang, X. Deconstorm: software for analyzing storm data (2012). URL `http://zhuang.harvard.edu/decon_storm.html`.

[14] Cox, S. *et al.* Bayesian localization microscopy reveals nanoscale podosome dynamics. *Nature Methods* **9**, 195–200 (2012).

[15] Yahiatene, I., Hennig, S., Müller, M. & Huser, T. Entropy-based super-resolution imaging (ESI): From disorder to fine detail. *ACS Photonics* **2**, 1049–1056 (2015).

[16] Dertinger, T., Colyer, R., Iyer, G., Weiss, S. & Enderlein, J. Fast, background-free, 3d super-resolution optical fluctuation imaging (SOFI). *Proceedings of the National Academy of Sciences* **106**, 22287–22292 (2009).
